# E6-mediated activation of JNK drives EGFR signalling to promote proliferation and viral oncoprotein expression in cervical cancer

**DOI:** 10.1038/s41418-020-00693-9

**Published:** 2020-12-10

**Authors:** Ethan L. Morgan, James A. Scarth, Molly R. Patterson, Christopher W. Wasson, Georgia C. Hemingway, Diego Barba-Moreno, Andrew Macdonald

**Affiliations:** 1 School of Molecular and Cellular Biology, Faculty of Biological Sciences, University of Leeds, Leeds, West Yorkshire LS2 9JT UK; 2 Astbury Centre for Structural Molecular Biology, University of Leeds, Leeds, West Yorkshire LS2 9JT UK; 3 Present Address: Tumor Biology Section, Head and Neck Surgery Branch, National Institute of Deafness and Other Communication Disorders, National Institute of Health, Bethesda, MD USA; 4 Present Address: Leeds Institute of Rheumatic and Musculoskeletal Medicine, School of Medicine, University of Leeds, St-James University Teaching Hospital, Leeds, West Yorkshire UK

**Keywords:** Cancer microenvironment, Oncogenes

## Abstract

Human papillomaviruses (HPV) are a major cause of malignancy worldwide, contributing to ~5% of all human cancers including almost all cases of cervical cancer and a growing number of ano-genital and oral cancers. HPV-induced malignancy is primarily driven by the viral oncogenes, E6 and E7, which manipulate host cellular pathways to increase cell proliferation and enhance cell survival, ultimately predisposing infected cells to malignant transformation. Consequently, a more detailed understanding of viral-host interactions in HPV-associated disease offers the potential to identify novel therapeutic targets. Here, we identify that the c-Jun N-terminal kinase (JNK) signalling pathway is activated in cervical disease and in cervical cancer. The HPV E6 oncogene induces JNK1/2 phosphorylation in a manner that requires the E6 PDZ binding motif. We show that blockade of JNK1/2 signalling using small molecule inhibitors, or knockdown of the canonical JNK substrate c-Jun, reduces cell proliferation and induces apoptosis in cervical cancer cells. We further demonstrate that this phenotype is at least partially driven by JNK-dependent activation of EGFR signalling via increased expression of EGFR and the EGFR ligands EGF and HB-EGF. JNK/c-Jun signalling promoted the invasive potential of cervical cancer cells and was required for the expression of the epithelial to mesenchymal transition (EMT)-associated transcription factor Slug and the mesenchymal marker Vimentin. Furthermore, JNK/c-Jun signalling is required for the constitutive expression of HPV E6 and E7, which are essential for cervical cancer cell growth and survival. Together, these data demonstrate a positive feedback loop between the EGFR signalling pathway and HPV E6/E7 expression, identifying a regulatory mechanism in which HPV drives EGFR signalling to promote proliferation, survival and EMT. Thus, our study has identified a novel therapeutic target that may be beneficial for the treatment of cervical cancer.

## Introduction

Persistent infection with human papillomavirus (HPV) is the underlying cause of most cervical cancers and several other anogenital and oropharngeal cancers [1]. These cancers are predominantly caused by HPV16 and HPV18; however, 13 other high-risk HPV types are also associated with cancer development [2]. HPV-induced transformation is primarily driven by three virus-expressed oncogenes: E5, E6 and E7. HPV E5 functions as an ion channel, induces EGFR signalling and promotes resistance to anti-PD-L1 immunotherapy [3–6]. E6 and E7 are the primary drivers of viral oncogenesis; in addition to their well-characterised inactivation of the p53 and pRb tumour suppressors [7, 8], they regulate a multitude of signalling pathways that contribute to transformation [9–15].

Mitogen-activated protein kinases (MAPK) convert extracellular stimuli into a range of cellular responses [16]. The MAPKs extracellular signal-regulated kinase (ERK) and p38 have been shown to have important roles in the HPV life cycle and in HPV-induced transformation [17–19]. In contrast, less is known about the role of the c-Jun N-terminal kinases (JNK).

JNKs consist of 10 isoforms, generated via alternative splicing from three genes: *JNK1* and *JNK2* (four isoforms of each) are ubiquitously expressed, whereas *JNK3* (two isoforms) is mainly expressed in the brain, heart and testes [20]. JNK signalling can regulate tumour suppressive and oncogenic functions [21]. In combination with oncogenic Ras, JNK can either suppress or promote oncogenesis [22–24]. JNK can also promote malignancy in combination with PTEN loss or expression of the BCR-Abl oncogene [25, 26]. Interestingly, JNK1 and JNK2 can have opposing roles during tumour development, making JNK signalling not only complex but also tissue-type specific [27, 28].

We previously observed increased JNK1/2 phosphorylation in primary normal human keratinocytes (NHK) stably harbouring the HPV18 genome [29]; however, it is not known if JNK contributes to transformation in HPV-associated cancers. In this study, we demonstrate that phosphorylation of JNK1/2 and its substrate c-Jun is enhanced in HPV+ cervical cancer. We show that JNK1/2 signalling is critical for the proliferative and invasive properties of cervical cancer cells and is required for basal and growth factor-induced viral oncogene expression. Together, we identify a host signalling pathway that plays a key role in cervical cancer progression that could potentially serve as a therapeutic target for HPV-associated cancers.

## Results

### c-Jun N-terminal kinase 1/2 (JNK1/2) phosphorylation is increased in cervical cancer

**Fig. 1:**
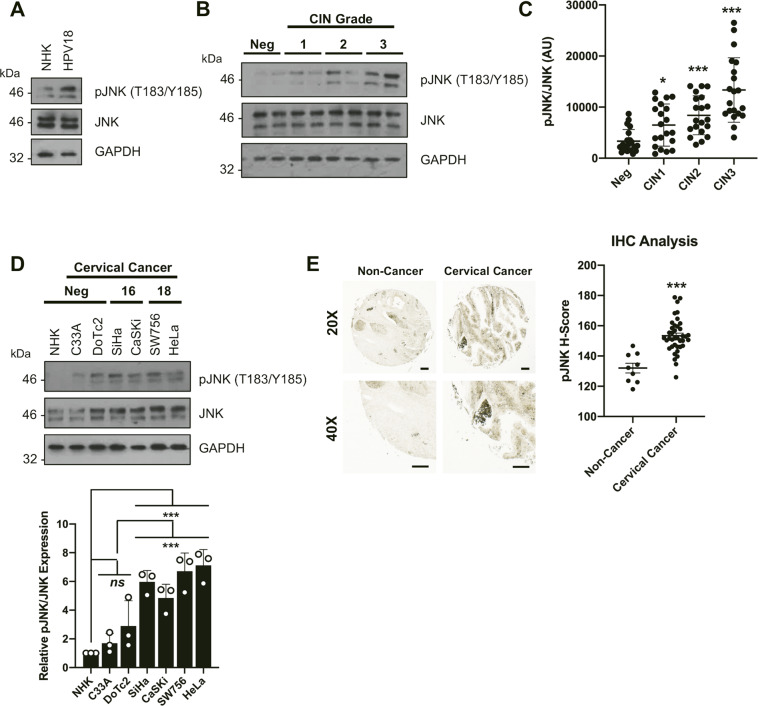
c-Jun N-terminal kinases are significantly phosphorylated in HPV+ cervical cancer. **A** Representative western blot of normal human keratinocytes (NHK) and NHKs containing the HPV18 genome (HPV18) analysed for phosphorylated JNK1/2 and total JNK expression. GAPDH served as a loading control. Data shown are representative of at least three independent experiments. **B** Representative western blot from cytology samples of CIN lesions of increasing grade analysed for phosphorylated JNK1/2 and total JNK expression. GAPDH served as a loading control. **C** Scatter plot of densitometry analysis of a panel of cytology samples. Twenty samples from each clinical grade (Neg, CIN 1–3) were analysed by western blot and densitometry analysis was performed for phosphorylated JNK1/2 and total JNK expression. **D** Representative western blot analysis of a panel of six cervical cancer cell lines—two HPV− (C33A and DoTc2 4510), two HPV16+ (SiHa and CaSKi) and HPV18+ (SW756 and HeLa)—and NHK cells for phosphorylated JNK1/2 and total JNK. GAPDH served as a loading control. Densitometry analysis of phosphorylated JNK1/2, normalised to total JNK and GAPDH expression, from three independent experiments is shown below. **E** Representative results of immunohistochemical staining for phosphorylated JNK1/2 expression in cervical cancer tissues and adjacent non-tumour tissues. Images were acquired using identical exposure times. Scale bar, 50 μm. Statistical analysis for phosphorylated JNK1/2 expression in cervical cancer tissues (*n* = 39) compared with non-tumour tissues (*n* = 9) is shown on the right. Each section was stained and analysed in duplicate. Phosphorylated JNK1/2 was quantified in an automated fashion using the IHC Profiler Plug-in for ImageJ. Error bars represent the mean +/− standard deviation. *P < 0.05, **P < 0.01, ***P < 0.001 (Student’s *t*-test).

We have published that JNK1/2 phosphorylation is increased in NHKs containing the HPV18 genome when compared to donor-matched control primary cells ([29]; Fig. 1A). We therefore investigated the role of JNK signalling in HPV-associated disease. JNK1/2 phosphorylation was analysed in cytology samples from a cohort of HPV16+ patients with cervical intraepithelial neoplasia (CIN); CIN1 represents a transient HPV infection with mild dysplasia, while CIN3 represents severe dysplasia [30]. Cytology samples from healthy, HPV-negative (HPV−) patients were used as controls. JNK1/2 phosphorylation increased during progression through CIN1 to CIN3, whilst total JNK1/2 expression remained constant (Fig. 1B; quantified in Fig. 1C). We next examined JNK1/2 phosphorylation in a panel of cervical cancer cell lines. Compared to NHKs, JNK1/2 phosphorylation was higher in all cervical cancer cells and was highest of all in HPV-positive (HPV+) cells (Fig. 1D). To determine if this extended to cervical cancer tissue, we performed immunohistochemistry (IHC) on a cervical cancer tissue microarray (TMA). JNK1/2 phosphorylation was significantly higher in the cervical cancer tissue when compared to control tissue (Fig. 1E). These data demonstrate that JNK1/2 phosphorylation is increased during cervical disease progression and in cervical cancer.

### JNK1/2 are required for c-Jun/AP-1 activity in cervical cancer

JNKs accomplish many of their functions by phosphorylating and activating Activator Protein-1 (AP-1) transcription factors [31]. We focused on Jun proteins, as these are commonly associated with tumourigenesis, particularly in cancers arising from keratinocytes, and c-Jun is a major cellular substrate of JNK [32, 33]. We observed increased c-Jun and JunD transcript and protein levels in HPV18 containing keratinocytes (Supplementary Fig. 1A, B). This increase was also seen in HPV+ cancer cells (Supplementary Fig 1C, D). In contrast, *JUNB* expression was unchanged (Supplementary Fig. 1A and C). Finally, using data from The Cancer Genome Atlas (TCGA), we observed that *JUN* expression significantly correlated with reduced survival in cervical cancer patients, whereas *JUND* expression did not (Supplementary Fig. 1E). We therefore focused on c-Jun as a potential downstream target of JNK1/2 in subsequent studies.

**Fig. 2:**
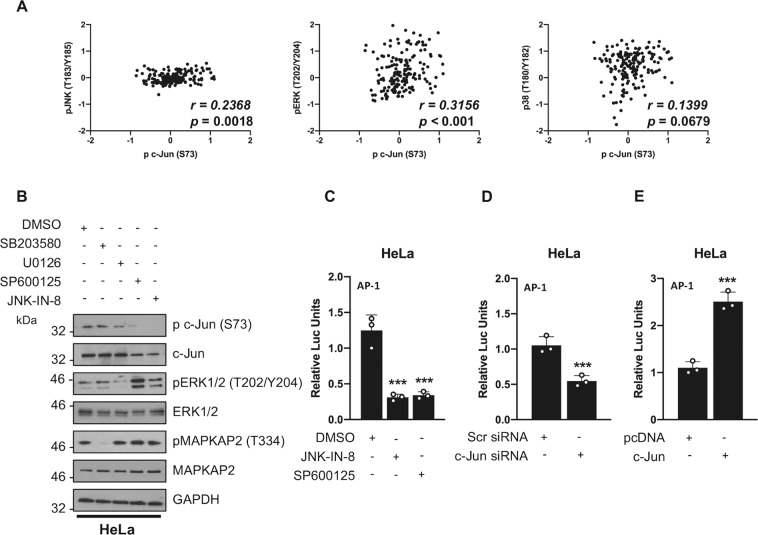
JNK activity is required for c-Jun/AP-1 activity in cervical cancer. **A** Scatter plot of expression levels of c-Jun phosphorylation and JNK phosphorylation, ERK phosphorylation and p38 phosphorylation as analysed using the MD Anderson public RPPA data set. The correlation coefficient (*r*) was calculated using Spearman’s rank analysis. **B** Representative western blot of HeLa cells treated with the p38 inhibitor SB203580 (10 µM), the MEK1/2 inhibitor U0126 (10 µM), or the JNK inhibitors SP600125 (10 µM) and JNK-IN-8 (3 µM). Lysates were analysed for phosphorylated and total c-Jun, phosphorylated and total ERK and phosphorylated and total MAPKAP2. GAPDH served as a loading control. Data shown are representative of at least three independent experiments. **C** Representative luciferase reporter assay from HeLa cells transfected with an AP-1 luciferase reporter, in the presence or absence of JNK-IN-8 (3 µM) or SP600125 (10 µM). Promoter activity was measured using a dual-luciferase system. Data are presented as relative to the DMSO control. **D** Luciferase reporter assay from HeLa cells co-transfected with an AP-1 luciferase reporter and pool of siRNA against the JNK substrate c-Jun. Promoter activity was measured using a dual-luciferase system. Data are presented as relative to the scrambled siRNA transfection control. **E** Representative luciferase reporter assay from HeLa cells co-transfected with an AP-1 luciferase reporter and c-Jun. Promoter activity was measured using a dual-luciferase system. Data are presented as relative to the pcDNA transfection control. Error bars represent the mean +/− standard deviation of a minimum of three biological repeats. *P < 0.05, **P < 0.01, ***P < 0.001 (Student’s *t*-test).

The transcriptional activity of c-Jun is regulated by Ser63/Ser73 phosphorylation [34]. ERK, JNK and p38 are all reported to phosphorylate these sites [34–36]. Using a reverse-phase protein array (RPPA) data set [37, 38], phosphorylation of ERK1/2 and JNK1/2, but not p38, significantly correlated with c-Jun phosphorylation in cervical cancer (Fig. 2A). To validate this, we used specific inhibitors targetting MEK1/2 (which inhibits ERK1/2), JNK1/2 and p38 and assessed c-Jun phosphorylation. Inhibition of p38 had no effect on c-Jun phosphorylation, whilst ERK1/2 inhibition led to a small (~30%) decrease in c-Jun phosphorylation. Crucially, inhibition of JNK1/2 using two chemically distinct inhibitors resulted in a ~70% and ~90% reduction, suggesting that JNK1/2 are the primary kinases required for c-Jun phosphorylation in HPV+ cervical cancer cells (Fig. 2B). Pharmacological inhibition of JNK1/2 also resulted in a ~75% reduction in AP-1 transcriptional activity (Fig. 2C). To investigate if c-Jun was required for AP-1 signalling in these cells, we depleted c-Jun expression which resulted in a ~50% reduction in AP-1 activity (Fig. 2D). Over-expression of c-Jun resulted in a ~2.5-fold increase in AP-1 activity (Fig. 2E), together suggesting that JNK/c-Jun signalling drives AP-1 activity in HPV+ cervical cancer cells.

### HPV E6 induces JNK1/2 phosphorylation via the PDZ-binding motif (PBM)

**Fig. 3:**
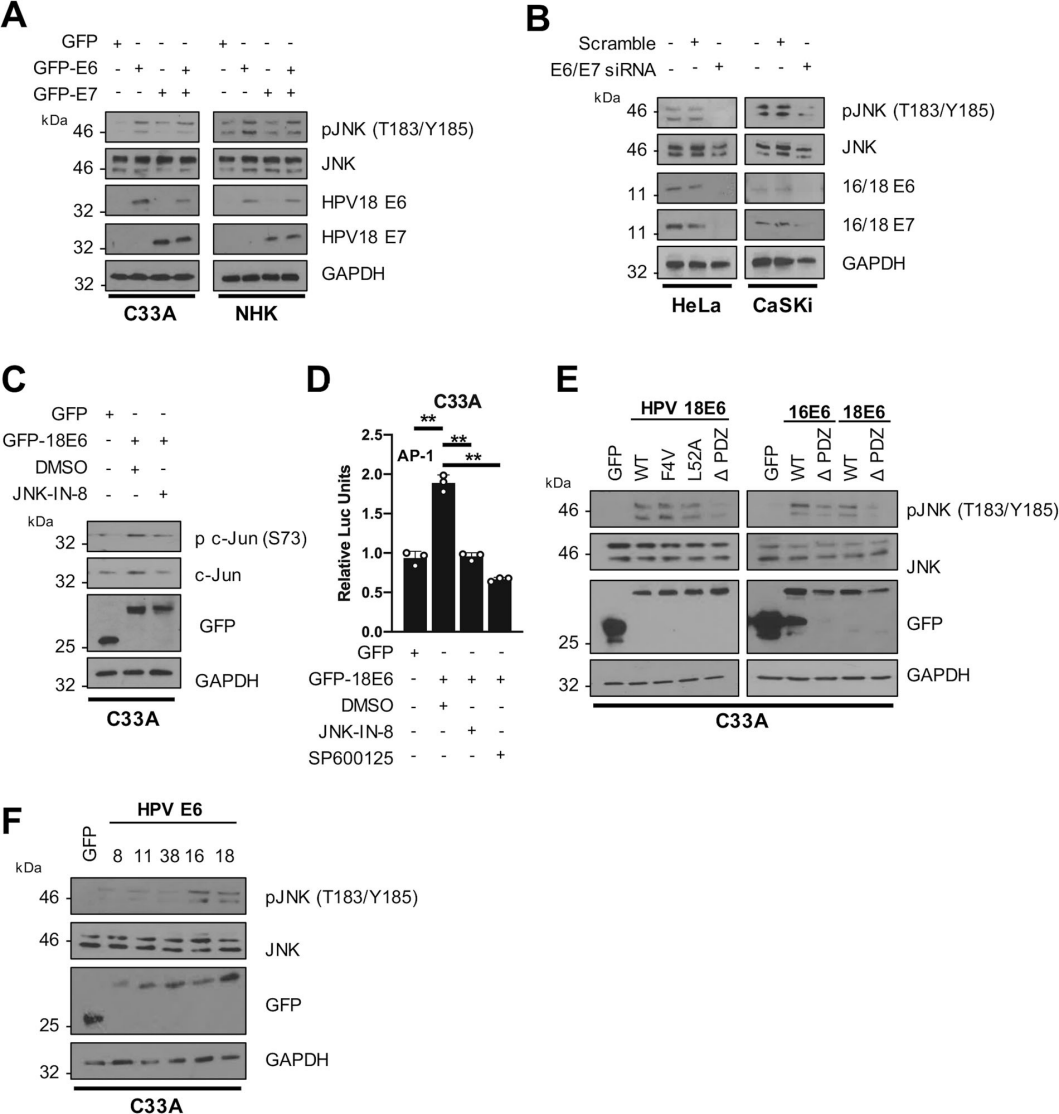
HPV E6 induces JNK phosphorylation via its PDZ-binding domain. **A** Representative western blot of C33A and NHK cells transiently transfected with GFP or GFP tagged HPV18 E6, E7 and E6/E7 and analysed for phosphorylated JNK1/2 and total JNK expression. Expression of HPV oncoproteins was confirmed by fluorescence microscopy for GFP expression (not shown) and HPV18 E6 and HPV18 E7 specific antibodies. GAPDH served as a loading control. Data shown are representative of at least three independent experiments. **B** Representative western blot of HeLa and CaSKi cells transfected with a pool of specific siRNAs against HPV18 E6/E7 or HPV16 E6/E7 respecitvely and analysed for phosphorylated JNK1/2 and total JNK expression. GAPDH served as a loading control. Data shown are representative of at least three independent experiments. **C** Representative western blot of C33A cells co-transfected with GFP or GFP tagged E6, in the presence or absence of JNK-IN-8 (3 µM). Lysates were analysed for phosphorylated c-Jun and total c-Jun expression. GAPDH served as a loading control. Data shown are representative of at least three independent experiments. **D** Luciferase reporter assay from C33A cells co-transfected with GFP or GFP tagged E6, and an AP-1 luciferase reporter, in the presence or absence of JNK-IN-8 (3 µM) or SP600125 (10 µM). Promoter activity was measured using a dual-luciferase system. Data are presented as relative to the GFP transfection control. **E** Representative western blot of C33A cells transiently transfected with GFP tagged E6 and E6 mutants and analysed for phosphorylated JNK and total JNK expression. Expression of HPV E6 and mutants was confirmed using a GFP antibody and GAPDH served as a loading control. Data shown are representative of at least three independent experiments. **F** Representative western blot of C33A cells co-transfected with GFP tagged E6 from different HPV types and analysed for phosphorylated JNK and total JNK expression. Expression of HPV E6 proteins was confirmed using a GFP antibody and GAPDH served as a loading control. Data shown are representative of at least three independent experiments. Error bars represent the mean +/− standard deviation of a minimum of three biological repeats. *P < 0.05, **P < 0.01, ***P < 0.001 (Student’s *t*-test).

Expression of HPV18 E6 in C33A cells and NHKs increased JNK1/2 phosphorylation (Fig. 3A), whilst E7 expression only slightly increased JNK1/2 phosphorylation and did not significantly enhance JNK1/2 phosphorylation when co-expressed with E6. Further, depletion of endogenous E6/E7 by siRNA reduced the level of JNK1/2 phosphorylation in both HeLa and CaSKi cells (Fig. 3B). Given that HPV E6 induced JNK1/2 phosphorylation, we investigated if E6 was responsible for the observed JNK-dependent AP-1 activity. Expression of HPV18 E6 increased c-Jun phosphorylation and enhanced AP-1 activity (Fig. 3C, D). Importantly, the increase in c-Jun phosphorylation and AP-1 activity was abolished in cells treated with inhibitors of JNK1/2.

To gain a greater insight into how HPV E6 increased JNK1/2 phosphorylation, we utilised a number of well-characterised E6 mutants. Both HPV18 E6-F4V, which cannot induce p53 degradation [39], and E6-L52A, which inhibits the interaction with E6AP [40], increased JNK1/2 phosphorylation to levels comparable with wild type (WT) E6. In contrast, an E6 mutant lacking the carboxyl-terminal PBM (ΔPDZ; [41]) failed to increase JNK1/2 phosphorylation, suggesting that E6 binding to PDZ-domain-containing proteins is required for JNK1/2 phosphorylation (Fig. 3E). This observation was also confirmed with the HPV16 E6 protein (Fig. 3E). Only E6 proteins from high-risk HPV types contain a PBM. In agreement with this, expression of E6 from the α-genus low-risk types 11, or the β-genus types HPV8 and HPV38 did not increase JNK1/2 phosphorylation (Fig. 3F). Together, these data demonstrate that high-risk E6 induces JNK1/2 phosphorylation in a PBM-dependent manner.

### Inhibition of JNK activity is detrimental to HPV+ cervical cancer cell proliferation

**Fig. 4:**
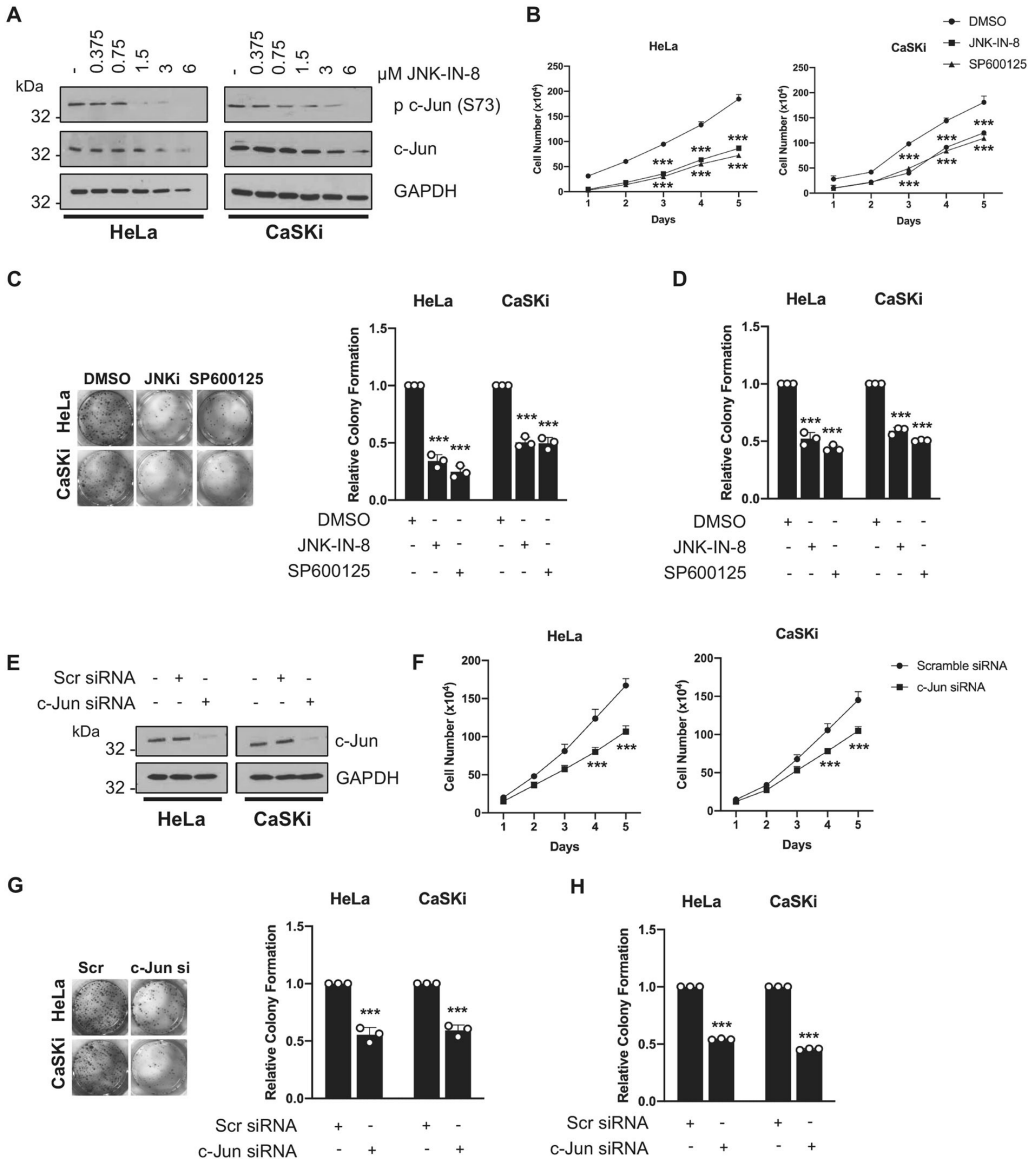
Inhibition of JNK/c-Jun signalling impaires the proliferation of HPV+ cervical cancer cells. **A** Representative western blots of HeLa and CaSKi cells treated with increasing doses of JNK-IN-8. Lysates were analysed for the phosphorylation and expression of the JNK substrate c-Jun. GAPDH was used as a loading control. Data shown are representative of at least three independent experiments. **B** Growth curve analysis of HeLa and CaSKi cells treated with JNK-IN-8 (3 µM) or SP600125 (10 µM). **C** Colony formation assay (anchorage-dependent growth) of HeLa and CaSKi cells treated with JNK-IN-8 (3 µM) or SP600125 (10 µM). **D** Soft agar assay (anchorage-independent growth) of HeLa and CaSKi cells treated with JNK-IN-8 (3 µM) or SP600125 (10 µM). **E** Representative western blots of HeLa and CaSKi transfected with a pool of siRNA against c-Jun. Lysates were analysed for the expression of c-Jun. GAPDH was used as a loading control. Data shown are representative of at least three independent experiments. **F** Growth curve analysis of HeLa and CaSKi cells transfected with a pool of siRNA against c-Jun. **G** Colony formation assay (anchorage-dependent growth) of HeLa and CaSKi cells transfected with a pool of siRNA against the JNK substrate c-Jun. **H** Soft agar assay (anchorage-independent growth) of HeLa and CaSKi cells transfected with a pool of siRNA against c-Jun. Error bars represent the mean +/− standard deviation of a minimum of three biological repeats. *P < 0.05, **P < 0.01, ***P < 0.001 (Student’s *t*-test).

JNK signalling can promote or inhibit cell proliferation, depending on the cellular context [22, 23]. To assess the impact of JNK/c-Jun activity on cell proliferation, we treated HeLa and CaSKi cells with increasing doses of the JNK1/2 inhibitors JNK-IN-8 and SP600125 [42, 43]. Inhibition of JNK1/2 resulted in a dose-dependent loss of c-Jun phosphorylation (Fig. 4A and Supplementary Fig. 2A). There was also a reduction in c-Jun expression, as c-Jun positively regulates its own transcription [44]. Inhibition of JNK1/2 led to a significant decrease in cell growth (Fig. 4B) and anchorage-dependent and anchorage-independent colony formation (Fig. 4C, D). Conversely, inhibition of JNK1/2 in HPV- C33A cells had minimal impact on cell growth or colony formation, despite a dose-dependent loss of c-Jun phosphorylation and protein expression (Supplementary Fig. 4A–D), suggesting that HPV+ cervical cancer cells are more sensitive to the effects of JNK1/2 inhibition, corresponding with higher levels of JNK1/2 phosphorylation.

Previous studies have demonstrated distinct effects of JNK1 and JNK2 in tumourigenesis [45, 46]. To investigate the contribution of each JNK gene, we utilised dominant-negative JNK1 and JNK2 mutants, in which the phosphorylation motif TPY is mutated to APF, resulting in JNK proteins that cannot be activated or phosphorylate c-Jun (Supplementary Fig. 3A; [47]). We found that both JNK1 and JNK2 are required for cell growth (Supplementary Fig. 3B) and colony formation (Supplementary Fig. 3C–D). These data demonstrate that the activity of both JNK1 and JNK2 is required for the proliferative ability of HPV+ cervical cancer cells.

### Active c-Jun/AP-1 is required for the proliferation of HPV+ cervical cancer cells

To confirm whether the JNK-dependent cell proliferation we observed was mediated by c-Jun/AP-1 signalling, we either depleted or over-expressed c-Jun (Fig. 4E and Supplementary Fig. 5A). Depletion of c-Jun significantly reduced cell growth (Fig. 4F) and colony formation (Fig. 4G, H). Conversely, over-expression of c-Jun enhanced cell growth and colony formation (Supplementary Fig. 5B-D), suggesting that c-Jun is required for the proliferation of HPV+ cervical cancer cells. In addition, we transfected cells with a dominant-negative JunD (ΔJunD) lacking a transcriptional activation domain that can dimerize with other AP-1 proteins and inhibit AP-1 activation (Supplementary Fig. 6A; [48]). ΔJunD expression abolished AP-1 activity in HeLa cells (Supplementary Fig. 6, B), and inhibited cell growth and colony formation (Supplementary Fig. 6C–E). These data suggest that JNK/c-Jun/AP-1 signalling is required for the proliferation of HPV+ cervical cancer cells.

### Inhibition of JNK/c-Jun induces G2/M phase accumulation and apoptosis in cervical cancer cells

We investigated if the reduced HPV+ cell proliferation caused by JNK inhibition was caused by modulation of the cell cycle. JNK1/2 inhibition led to a significant increase of cells in the G2/M phase, suggesting that JNK activity was required for progression from G2 into mitosis, or release from mitosis into G1 (Supplementary Fig. 7A). Depletion of c-Jun led to a similar effect on cell cycle progression (Supplementary Fig. 7D).

Interestingly, inhibition of JNK1/2 or depletion of c-Jun also resulted in a significant increase in the proportion of cells in sub-G1, which is indicative of apoptosis (Supplementary Fig. 7A and D). We performed Annexin V assays and demonstrated that inhibition of JNK/c-Jun by either approach led to an increase in early and late apoptosis, demonstrating that JNK signalling is required for cell survival in HPV+ cervical cancer cells (Supplementary Fig. 7, B and E). Mechanistically, we demonstrated that loss of JNK activity or c-Jun expression correlated with the activation of caspase 3, increasing the proteolytic cleavage of PARP (Supplementary Fig. 7C and F). Together, these data suggest that JNK/c-Jun signalling is required for cell cycle progression and survival in HPV+ cervical cancer cells.

### JNK/c-Jun activity is required for cell migration/invasion and regulates epithelial to mesenchymal transition (EMT) in cervical cancer cells

**Fig. 5:**
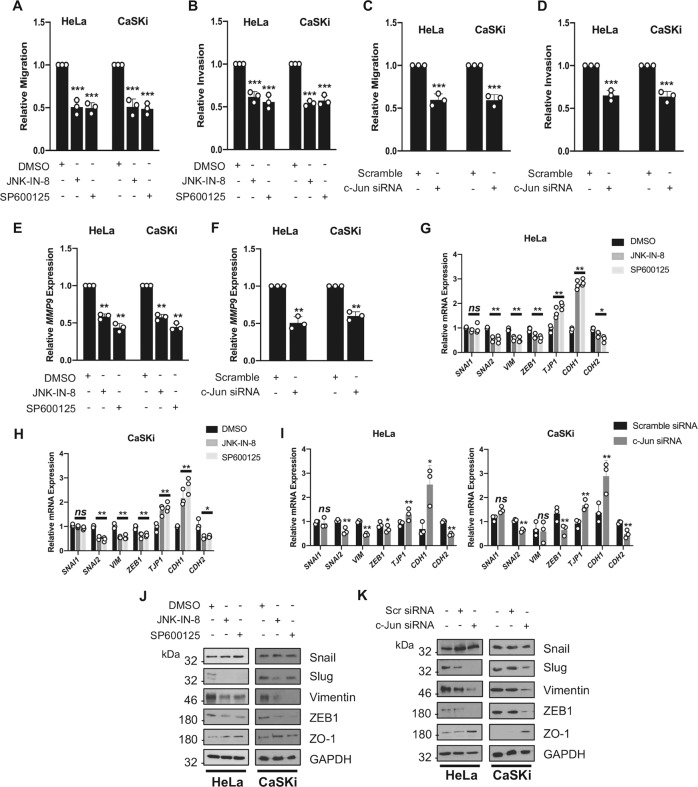
JNK/c-Jun activity is required for cell migration/invasion and regulates epithelial to mesenchymal transition (EMT). **A** Transwell® migration assay of HeLa and CaSKi cells treated with JNK-IN-8 (3 µM) or SP600125 (10 µM). The average number of invaded cells per field was calculated from five representative fields per experiment. **B** Transwell® invasion assay of HeLa and CaSKi cells treated with JNK-IN-8 (3 µM) or SP600125 (10 µM). The average number of invaded cells per field was calculated from five representative fields per experiment. **C** Transwell® migration assay of HeLa and CaSKi cells after transfection of a pool of siRNA against c-Jun. The average number of invaded cells per field was calculated from five representative fields per experiment. **D** Transwell® invasion assay of HeLa and CaSKi cells after transfection of a pool of siRNA against c-Jun. The average number of invaded cells per field was calculated from five representative fields per experiment. **E** qPCR analysis of *MMP9* mRNA expression HeLa and CaSKi cells treated with JNK-IN-8 (3 µM) or SP600125 (10 µM). *U6* was used as a loading control. **F** qPCR analysis of *MMP9* mRNA expression HeLa and CaSKi cells cells after transfection of a pool of siRNA against c-Jun. *U6* was used as a loading control. **G** qPCR analysis of genes involved in EMT in HeLa cells treated with JNK-IN-8 (3 µM) or SP600125 (10 µM). *U6* was used as a loading control. **H** qPCR analysis of genes involved in EMT in CaSKi cells treated with JNK-IN-8 (3 µM) or SP600125 (10 µM). *U6* was used as a loading control. **I** qPCR analysis of genes involved in EMT in HeLa and CaSKi cells after transfection of a pool of siRNA against c-Jun. *U6* was used as a loading control. **J** Representative western blots of HeLa and CaSKi treated JNK-IN-8 (3 µM) or SP600125 (10 µM). Lysates were probed for the expression of Slug, Snail, Vimentin, ZO-1 and ZEB1. GAPDH was used as a loading control. Data shown are representative of at least three independent experiments. **K** Representative western blots of HeLa and CaSKi after transfection of a pool of siRNA against c-Jun. Lysates were probed for the expression of Slug, Snail, Vimentin, ZO-1 and ZEB1. GAPDH was used as a loading control. Data shown are representative of at least three independent experiments. Error bars represent the mean +/− standard deviation of a minimum of three biological repeats. *P < 0.05, **P < 0.01, ***P < 0.001 (Student’s *t*-test).

Despite current survival rates demonstrating that existing treatments can be successful in cervical cancer (50–60% with radiotherapy and concurrent cisplatin treatment), many cases develop local recurrences and metastasise [49]. Furthermore, epithelial-mesenchymal transition (EMT) has been shown to increase cervical cancer progression via increased cell migration and invasion [50]. JNK signalling is a key driver of cell invasion in many cancers [51, 52]. We therefore investigated if JNK/c-Jun signalling contributed to the invasive phenotype of cervical cancer cells. Inhibition of JNK1/2 or depletion of c-Jun significantly reduced both the migration and invasion of cervical cancer cells through a Transwell® chamber membrane (Fig. 5A–D).

EMT and associated extracellular matrix remodelling are key processes enabling cancer cell invasion. JNK1/2 inhibition or c-Jun depletion reduced expression of matrix metalloprotease (MMP)-9, a crucial enzyme involved in cancer cell invasion (Fig. 5E, F). In addition, the expression of the EMT-associated transcription factors Slug (*SNAI2)* and ZEB1 (*ZEB1)*, but not Snail (*SNAI1*), and the mesenchymal markers N-cadherin (*CDH2*) and vimentin (*VIM*), was also reduced (Fig. 5G–K). Conversely, expression of the epithelial markers E-cadherin (*CDH1*) and ZO-1 (*TJP1*) was increased, suggesting that JNK/c-Jun activity is required for the invasive potential of cervical cancer cells, potentially through the regulation of EMT-associated proteins.

### JNK activity promotes EGFR signalling to regulate proliferation

In keratinocytes, proliferation is often mediated via binding of the epidermal growth factor (EGF)-family of ligands to the EGF receptor (EGFR) [32, 53, 54]. Several EGF-family ligands, as well as the EGFR itself, are regulated by c-Jun [55–57]. As EGFR signalling can be regulated by HPV E6 [58], we investigated whether the EGFR was required for the enhanced JNK activity observed. For this, HeLa cells were serum starved and treated with EGF (Supplementary Fig. 8). EGF treatment caused rapid and transient EGFR autophosphorylation (Y1068) and rapidly induced JNK1/2 phosphorylation, which peaked 10 min post-treatment and was undetectable 30 min post-treatment. This was followed by phosphorylation of the JNK substrates c-Jun and JunD, which started 15 min post treatment and peaked 45 min post treatment. By 1 h, an increase in both c-Jun and JunD protein expression was observed. These data show that EGF activated the JNK/c-Jun signalling pathway in cervical cancer cells.

**Fig. 6:**
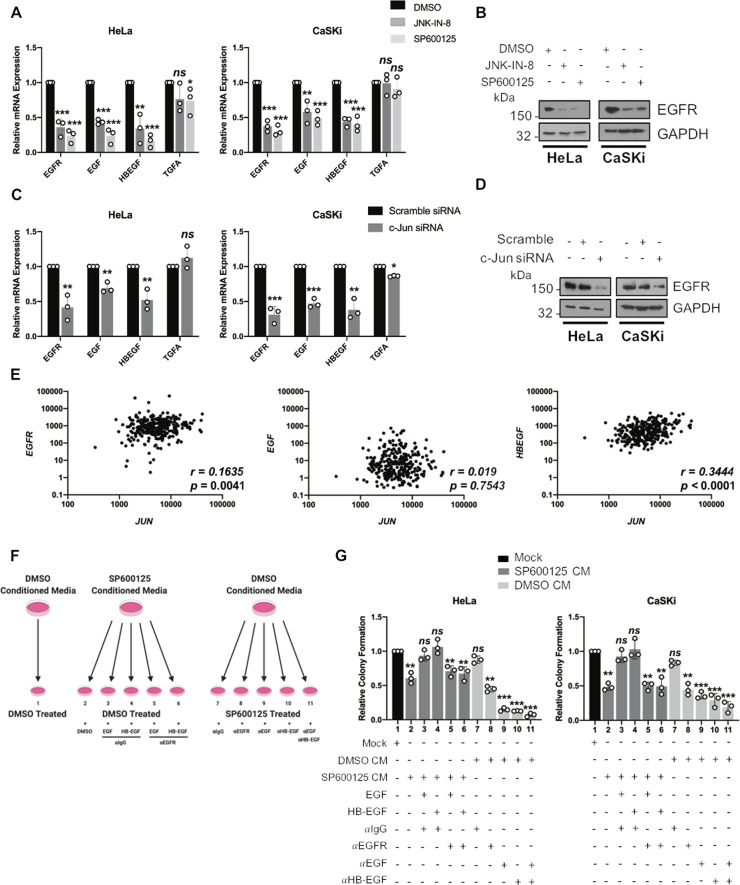
Regulation of EGFR signalling contributes to JNK mediated proliferation. **A** qPCR analysis of *EGFR* and EGFR ligand expression in HeLa and CaSKi cells treated with JNK-IN-8 (3 µM) or SP600125 (10 µM) for 48 h. *U6* was used as a loading control. **B)** Representative western blots of HeLa and CaSKi cells treated with JNK-IN-8 (3 µM) or SP600125 (10 µM) for the expression of EGFR for 48 h. Data shown are representative of at least three independent experiments. **C** qPCR analysis of *EGFR* and EGFR ligand expression in HeLa and CaSKi cells after transfection of a pool of siRNA against c-Jun for 72 h. *U6* was used as a loading control. **D** Representative western blot of HeLa and CaSKi cells after transfection of a pool of siRNA against c-Jun for the expression of EGFR for 72 h. Data shown are representative of at least three independent experiments. **E** Scatter plot of expression levels of *JUN* and *EGFR*, *EGF* and *HBEGF* as analysed using the MD Anderson public RPPA data set. The correlation coefficient (*r*) was calculated using Spearman’s rank analysis. **F** Schematic for conditioned media rescue assay. **G** Colony formation assay (anchorage-dependent growth) of HeLa and CaSKi treated with SP600125 (10 µM) or DMSO control for 48 h. SP600125 was subsequently washed out and control or SP600125-treated cells were grown in conditioned media from cells treated with DMSO or SP600125. In addition, cells were also grown in SP600125 conditioned media containing EGF, HB-EGF, or DMSO conditioned media with or without neutralising EGFR, EGF or HB-EGF antibodies. Colonies were stained and counted after 14 days. Error bars represent the mean +/− standard deviation of a minimum of three biological repeats. *P < 0.05, **P < 0.01, ***P < 0.001 (Student’s *t*-test).

We next investigated if the JNK/c-Jun pathway regulated EGFR signalling in cervical cancer cells. First, we inhibited JNK activity and used qRT-PCR to measure the expression of *EGFR* and the EGFR ligands EGF, transforming growth factor α (TGFα) and heparin-binding EGF (HB-EGF). Inhibition of JNK activity significantly reduced *EGFR, EGF* and *HBEGF* expression, without affecting the expression of *TGFA* (Fig. 6A). This led to a loss of EGFR protein expression (Fig. 6B). Knockdown of c-Jun had similar effects (Fig. 6C, D), suggesting that JNK/c-Jun signalling regulated the expression of key EGFR signalling components in cervical cancer cells. Data from the TCGA demonstrated that c-Jun expression correlated with *EGFR*, *EGF* and *HBEGF* expression (Fig. 6E).

Next, we assessed if impaired EGFR signalling was responsible for the reduction in proliferation observed upon inhibition of the JNK/c-Jun pathway. To do this, we incubated cells previously treated with DMSO or SP600125 (as SP600125 is a reversible inhibitior [42]) in conditioned media from cells treated with DMSO or SP600125 (diagram in Fig. 6F). Incubation of DMSO treated cells with conditioned media taken from SP600125-treated cells significantly reduced colony formation (Fig. 6G, compare bar 2 to bar 1). To determine if this was due to impaired EGF/HB-EGF-EGFR signalling, we supplemented conditioned media from SP600125 cells with EGF or HB-EGF. The addition of either EGFR ligand restored colony formation to control levels (Fig. 6G, compare bars 3 and 4 with bar 1). We confirmed that the restoration of colony formation was due to EGFR signalling, as co-incubation of EGF or HB-EGF with a neutralising EGFR antibody nullified this effect (compare bars 3 and 4 with bars 5 and 6).

To confirm these findings, we incubated SP600125-treated cells, which exhibit reduced colony formation (Fig. 4C), with conditioned media from DMSO treated cells, which should contain normal levels of EGF and HB-EGF. In agreement with our previous data, this restored colony formation to control levels (Fig. 6G, compare bar 7 with bar 1). This supports the hypothesis that a soluble factor may rescue proliferation in cells in which JNK1/2 was inhibited. The addition of media containing a neutralising EGFR antibody failed to rescue colony formation, suggesting that EGFR signalling plays a role in the diminished proliferative ability of JNK-inhibited cervical cancer cells (Fig. 6G, compare bar 8 with bar 7). Finally, to confirm if EGF and/or HB-EGF were the EGFR ligands implicated in the restoration of proliferation, we incubated conditioned media from DMSO treated cells with neutralising antibodies against EGF and/or HB-EGF. The addition of media containing either or both neutralising antibodies failed to rescue colony formation (Fig. 6G, compare bars 9, 10 and 11 with bar 7). Taken together, these data suggest that EGF/HB-EGF-EGFR signalling is required for the proliferative ability of HPV+ cervical cancer cells, and this signalling axis is reduced in cells in which JNK is inhibited.

### JNK mediated c-Jun activation plays a critical role in HPV oncogene expression

**Fig. 7:**
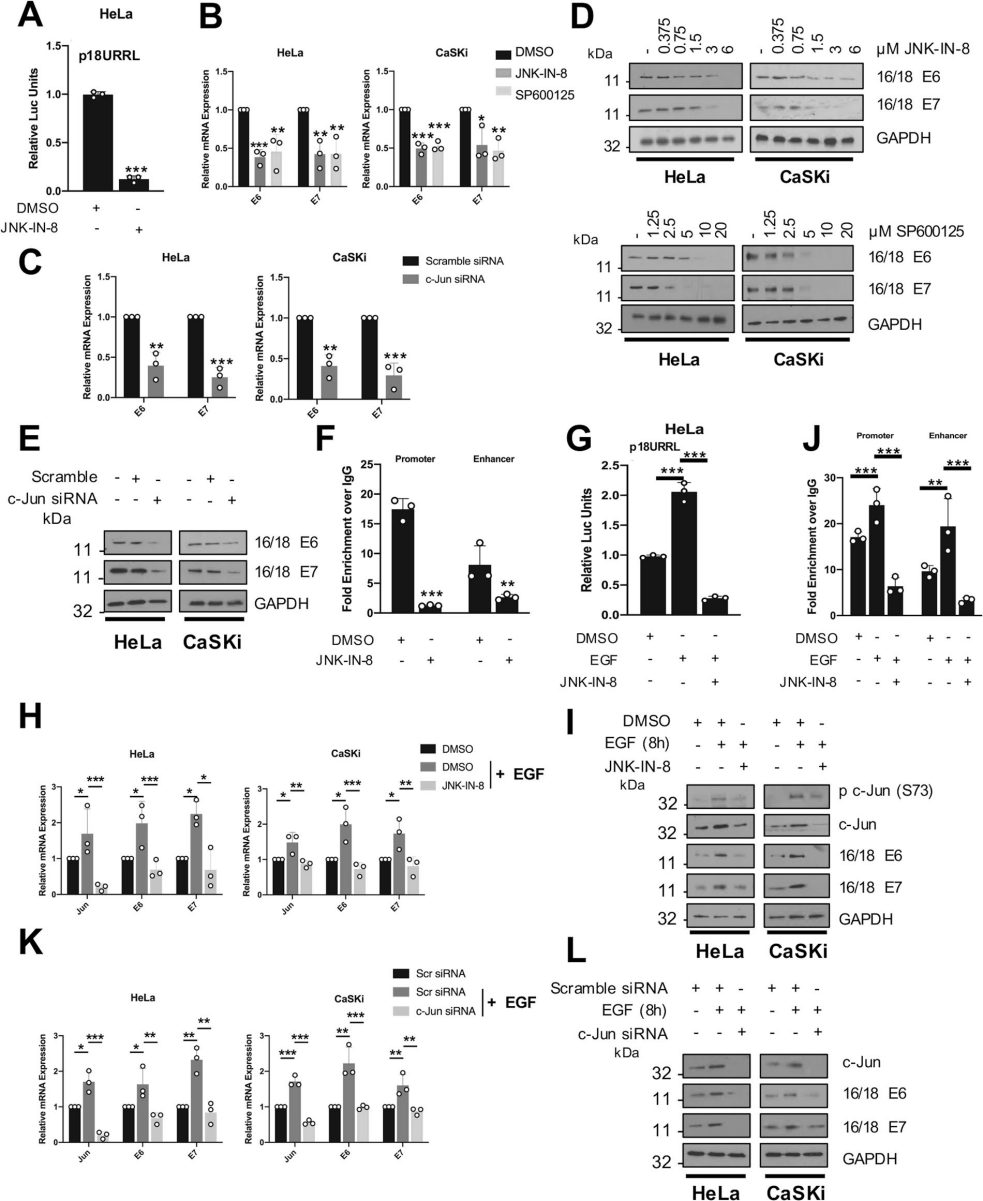
JNK mediated c-Jun activation plays a significant role in HPV oncogene expression. **A** Luciferase reporter assay from HeLa cells transfected with a HPV18 URR luciferase reporter with or without treatment with JNK-IN-8 (3 µM). Promoter activity was measured using a dual-luciferase system. Data are presented as relative to the DMSO control. **B** qPCR analysis of HPV 16/18 *E6* and *E7* mRNA expression in HeLa and CaSKi cells treated with JNK-IN-8 (3 µM). *U6* was used as a loading control. **C** qPCR analysis of HPV 16/18 *E6* and *E7* mRNA expression in HeLa and CaSKi cells after transfection of a pool of siRNA against c-Jun. *U6* was used as a loading control. **D** Representative western blots of HeLa and CaSKi treated with increasing doses of JNK-IN-8 (3 µM) or SP600125 (10 µM). Lysates were analysed for the expression of HPV 16/18 E6 and E7. GAPDH was used as a loading control. Data shown are representative of at least three independent experiments. **E** Representative western blots of HeLa and CaSKi after transfection of a pool of siRNA against c-Jun. Lysates were analysed for the expression of HPV 16/18 E6 and E7. GAPDH was used as a loading control. Data shown are representative of at least three independent experiments. **F** ChIP-qPCR analysis of c-Jun binding to the HPV18 URR in HeLa cells with or without JNK-IN-8 (3 µM). Chromatin was prepared from HeLa cells and c-Jun was immunoprecipitated using an anti-c-Jun antibody, followed by RT-qPCR using primers specific to AP-1 binding sites in the HPV18 URR enhancer or promoter region. c-Jun binding is presented as percentage of input chromatin. **G** Luciferase reporter assay from HeLa cells transfected with a HPV18 URR luciferase reporter and treated with EGF (50 ng/mL, 8 h) with or without treatment with JNK-IN-8 (3 µM). Promoter activity was measured using a dual-luciferase system. Data are presented as relative to the GFP transfected control. **H** qPCR analysis of HPV 16/18 *E6* and *E7* mRNA expression in HeLa and CaSKi cells treated with EGF (50 ng/mL, 8 h) with or without JNK-IN-8 (3 µM). *U6* was used as a loading control. **I** Representative western blots of HeLa and CaSKi treated with EGF (50 ng/mL, 8 h) with or without JNK-IN-8 (3 µM). Lysates were analysed for the expression of HPV 16/18 E6 and E7. GAPDH was used as a loading control. Data shown are representative of at least three independent experiments. **J** ChIP-qPCR analysis of c-Jun binding to the HPV18 URR in HeLa cells treated with EGF (50 ng/mL, 8 h), with or without JNK-IN-8 (3 µM). Chromatin was prepared from HeLa cells and c-Jun was immunoprecipitated using an anti-c-Jun antibody, followed by RT-qPCR using primers specific to AP-1 binding sites in the HPV18 URR enhancer or promoter region. c-Jun binding is presented as percentage of input chromatin. **K** qPCR analysis of HPV 16/18 *E6* and *E7* mRNA expression in HeLa and CaSKi cells treated with EGF (50 ng/mL, 8 h) after transfection of a pool of siRNA against c-Jun. *U6* was used as a loading control. **L** Representative western blots of HeLa and CaSKi treated with EGF (50 ng/mL, 8 h) after transfection of a pool of siRNA against c-Jun. Lysates were analysed for the expression of HPV E6 and E7. GAPDH was used as a loading control. Data shown are representative of at least three independent experiments. Error bars represent the mean +/− standard deviation of a minimum of three biological repeats. *P < 0.05, **P < 0.01, ***P < 0.001 (Student’s *t*-test).

The continued expression of the viral oncogenes E6 and E7 is required for the proliferation and survival of HPV-associated cancers [59]. Viral oncogene expression is regulated by host transcription factors through binding to the upstream regulatory region (URR) in the virus genome [60]. The URR contains AP-1 binding sites in both the promoter and enhancer regions that can bind c-Jun and induce E6/E7 expression [61–65]. The signalling that governs this AP-1 dependent induction of E6/E7 is unclear. To investigate if JNK signalling played a role, we assessed the activity of the viral URR using a reporter construct containing the HPV18 URR sequence [65]. Inhibition of JNK1/2 led to a ~90% reduction in URR-driven luciferase levels, suggesting that JNK signalling is critical for viral transcription (Fig. 7A). In addition, treatment of HeLa and CaSKi cells with JNK inhibitors resulted in a significant reduction in E6/E7 mRNA expression (Fig. 7B). Depletion of the JNK substrate c-Jun also reduced E6/E7 mRNA (Fig. 7C) and protein (Fig. 7D, E) expression.

ChIP analysis was used to confirm if JNK1/2 inhibition resulted in reduced c-Jun recruitment to the viral URR, utilising primers covering the AP-1 binding sites in both the viral promoter and enhancer. JNK inhibition led to a significant reduction in c-Jun recruitment to the AP-1 binding sites in both the viral promoter and enhancer (Fig. 7F).

As we had demonstrated a role for EGF signalling in JNK activity, we assessed if JNK was responsible for EGF-induced E6/E7 expression. Treatment of HeLa cells with EGF increased viral transcription and E6/E7 expression (Fig. 7G–I); critically, inhibition of JNK abolished this EGF-induced oncoprotein expression. Further, EGF stimulation increased binding of c-Jun to the viral URR in a JNK-dependent manner (Fig. 7J). As expected, depletion of c-Jun also led to a reduction in EGF-induced viral transcription and E6/E7 expression, demonstrating that c-Jun is required for the EGF-induced E6/E7 expression (Fig. 7K, L).

## Discussion

Despite the availability of preventative vaccines against HPV, there is a lack of specific therapies targetting HPV-associated malignancies. As such, there is a critical need for research into the fundamental biology of the virus-host interactions to identify host factors that are essential in HPV-associated cancers. Indeed, targetting host cell proteins with small molecule inhibitors or nucleic acid mimics has shown potential as antiviral therapies for a number of oncogenic viruses, including Kaposi’s sarcoma-associated herpesvirus (KSHV; [66]) and HPV [14, 67]. HPV manipulates signalling pathways which contribute to its oncogenic potential, including the JAK/STAT [13, 14, 29], PI3K/AKT [10, 68], Hippo [69] and MAPK pathways [17, 19]. Here, we show aberrant JNK signalling in HPV-containing keratinocytes and cervical cancer cells.

JNK activity can be either tumour suppressive or oncogenic, depending on the cellular context. For instance, JNK activity is associated with the induction of apoptosis and can suppress Ras-induced transformation in MEF xenografts [23]. In contrast, JNK is essential for transformation by oncogenic Ras in lung carcinoma and c-Met in mouse fibroblasts [22, 70]. Many of these pro-oncogenic functions of JNK are dependent on the c-Jun transcription factor [25, 32]. Thus, the function of JNK activity is cancer and cell-type specific. Using a combination of samples from patients with cervical disease and cervical cancer tissue, we show that JNK1/2 phosphorylation correlates with cervical disease progression and is increased in cervical cancer tissue and in HPV+ cervical cancer cell lines. This corresponds with previous data demonstrating high levels of JNK1/2 phosphorylation in cervical cancer [71]. Our data demonstrate that the increased JNK1/2 phosphorylation correlates with enhanced c-Jun phosphorylation and AP-1 activity, which is a key regulator of proliferation in many malignancies, including cervical cancer [72–74]. Importantly, we demonstrate that inhibition of JNK1/2 using small molecule inhibitors, inactive mutants and depletion of the JNK substrate c-Jun reduces cell proliferation and increases apoptosis.

We also observed that the inhibition of JNK1/2 in HPV+ cervical cancer cells increased the proportion of cells in the G2 phase of the cell cycle, consistent with previous reports [75]. This can lead to endoreduplication, mitotic spindle deformation and delayed apoptosis [76, 77]. The increase in sub-G1 DNA observed upon JNK1/2 inhibition in this study suggests that the mechanism of apoptosis observed in cervical cancer cells may be due to mitotic defects.

In addition, we demonstrate that the reduced proliferative capacity of HPV + cells observed upon JNK1/2 inhibition is at least partially due to a defect in EGFR signalling, which has previously been shown in skin tumours in which the *JUN* gene has been deleted [32, 53, 78, 79]. Here, disrupted JNK signalling reduced expression of *EGF*, *HBEGF* and *EGFR*. Experiments using conditioned media from control or JNK1/2 inhibitor-treated cells demonstrated that the proliferative defect upon JNK1/2 inhibition was rescued by the addition of soluble factors that were blocked by a neutralising EGFR antibody, suggesting they function through the EGFR. In confirmation of this, the addition of EGF or HB-EGF to conditioned media from JNK1/2-inhibited cells also rescued the proliferation defect. Neutralising antibodies against EGF or HB-EGF not only inhibited the restoration of proliferation, but inhibited proliferation to a greater extend that the SP600125-treated conditioned media. This suggests that these ligands may also signal via receptors other than the EGFR. EGF has been shown to activate ErbB4 homodimers, and both EGF and HB-EGF can activate HER2/ErbB4 heterodimer mediated mitogenic signalling [80]. Thus, the enhanced proliferation defect observed upon addition of EGF and/or HB-EGF neutralising antibodies may be due to the inhibition of mitogenic signalling through other ErbB receptors, not just the EGFR. Together, our data indicate that impaired EGFR/ErbB signalling contributes, in part, to the proliferative defects observed in HPV+ cervical cancer cells upon inhibition of JNK signalling.

HPV E6 has previously been shown to induce JNK/c-Jun phosphorylation in lung cancer cells [81]; however, a clear role for E6-mediated JNK/c-Jun signalling in cervical cancer cells is lacking. Our data demonstrate that the HPV E6 oncogene is responsible for JNK1/2 phosphorylation. We further demonstrate that this is dependent on the PBM found only in high-risk E6 proteins. As such, the induction of JNK1/2 phosphorylation may be an oncogenic function of E6. It is not clear how binding to cellular PDZ domains modulates JNK1/2 phosphorylation. Several PDZ-domain-containing targets of E6 are involved in cell polarity [82]; in combination with oncogenic Ras mutants, loss of the cell polarity proteins, and known E6 targets, Scribble and DLG1, require JNK1/2 activation for tumourigenesis [83]. In addition, a *Drosophila* model of E6-mediated malignancy demonstrated a key role for the polarity protein MAGI [84]. Thus, HPV E6-mediated degradation of cell polarity proteins may be required to induce JNK1/2 phosphorylation.

Constitutive expression of the viral oncogenes E6 and E7 is essential for cancer progression. However, our understanding of the regulation of viral oncogene expression by host cell factors is incomplete. Previous data have demonstrated that the viral URR contains transcription factor binding sites necessary for viral oncogene expression [60, 85]. Interestingly, two AP-1 binding sites have been identified in the HPV18 URR, one in the enhancer region and one in the promoter, which are important in viral transcription [65]. Here, we demonstrate that the JNK signalling pathway is critical for the expression of HPV E6/E7. Importantly, the JNK substrate c-Jun mediates the effect of JNK activity on viral transcription, as recruitment of c-Jun to the AP-1 binding sites in the URR is abolished upon JNK1/2 inhibition. Furthermore, E6/E7 expression has previously been demonstrated to be induced by EGFR signalling [86, 87]; our data demonstrate that this requires JNK activity.

**Fig. 8:**
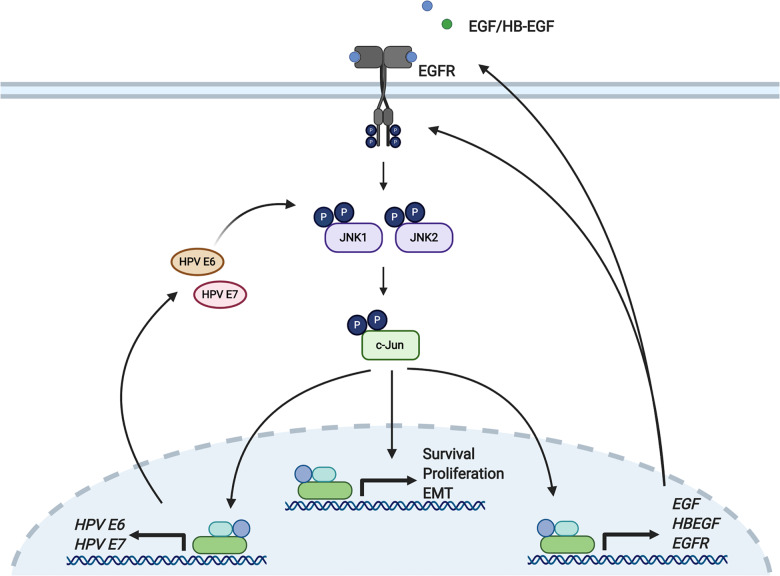
Schematic demonstrating E6-mediated JNK activation and downstream signalling. HPV E6 induces JNK1/2 phosphorylation and subsequent c-Jun/AP-1 activation. This drives the activation of genes involved in cell proliferation, such as EGFR and EGF ligands, which subsequently forms a positive feedback loop, driving JNK activation and proliferation. JNK signalling also drives the expression of genes involved in cell migration, invasion and EMT. Furthermore, the JNK/c-Jun/AP-1 signalling axis is required for basal and EGF-induced viral oncogene expression. Figure was created using BioRender.com.

Our study has uncovered the presence of a complex positive feedback loop in HPV+ cervical cancer cells (Fig. 8). In this pathway, HPV E6 activates JNK, which then phosphorylates and activates c-Jun, which transactivates host genes regulating cell growth and survival and can also bind AP-1 sequences in the HPV URR to drive expression of HPV E6/E7. Upregulated host factors include both the EGFR and its ligands EGF and HB-EGF. Signalling driven from the EGFR in turn activates JNK to potentiate the pathway. The existence of this signalling loop offers a potential target for therapy in HPV-associated cancers.

## Materials and methods

### Cervical cytology samples

Cervical cytology samples were obtained from the Scottish HPV Archive (http://www.shine/mvm.ed.ac.uk/archive.shtml). The East of Scotland Research Ethics Service has given generic approval to the Scottish HPV Archive as a Research Tissue Bank (REC Ref 11/AL/0174) for HPV related research on anonymised archive samples. Samples are available for the through application to the Archive Steering Committee (HPV Archive Application Ref 0034). RNA and protein were extracted from the samples using Trizol™ as described by the manufacturer (ThermoFisher Scientific, USA).

### TMA and immunohistochemistry

A cervical cancer TMA containing 39 cases of cervical cancer and 9 cases of normal cervical tissue (in duplicate) were purchased from GeneTex, Inc. (GTX21468). Slides were deparaffinised in xylene, rehydrated in a graded series of ethanol solutions and subjected to antigen retrieval in citric acid. Slides were blocked in normal serum and incubated in primary antibody (Phospho-SAPK/JNK (Thr183/Tyr185) (81E11; 4668, Cell Signalling Technology (CST))) overnight at 4 °C. Slides were then processed using the VECTASTAIN® Universal Quick HRP Kit (PK-7800; Vector Laboratories) as per the manufacturer’s instructions. Immunostaining was visualised using 3,3’-diaminobenzidine (Vector® DAB (SK-4100; Vector Laboratories)). Phospho-JNK immunostaining quantification was automated using ImageJ with the IHC Profiler plug-in [88]. Histology scores (H-score) were calculated based on the percentage of positively stained tumour cells and the staining intensity grade [89]. The staining intensities were classified into the following four categories: 0, no staining; 1, low positive staining; 2, positive staining; 3, strong positive staining. H score was calculated by the following formula: (3 × percentage of strong positive tissue) + (2 × percentage of positive tissue) + (percentage of low positive tissue), giving a range of 0–300.

### Cell culture

HeLa (HPV18+ cervical epithelial adenocarcinoma cells), SW756 (HPV18+ cervical squamous carcinoma cells), SiHa (HPV16+ cervical squamous carcinoma cells), CaSKi (HPV16+ cervical squamous carcinoma cells), C33A (HPV− cervical squamous carcinoma) and DoTc2-4510 (HPV−cervical squamous carcinoma) cells obtained from the ATCC were grown in DMEM supplemented with 10% Foetal Bovine Serum (FBS; ThermoFisher Scientific, USA) and 50 U/mL penicillin (Lonza, USA). Primary NHKs isolation from neonate foreskin tissues (ethical approval no. 06/Q1702/45) was performed in S. Roberts’ laboratory as described previously [90] and were maintained in serum-free medium (SFM; GIBCO, UK) supplemented with 25 µg/mL bovine pituitary extract (GIBCO) and 0.2 ng/mL recombinant EGF (GIBCO). All cells were cultured at 37 °C and 5% CO_2_. The generation HPV18 containing NHKs has been previously described [5].

All cells were negative for Mycoplasma during this investigation. Cell identity was recently confirmed by STR profiling.

### Plasmids, siRNA and reagents

Plasmids for HPV oncoproteins have been previously described [29]. pAP1-luc, a luciferase reporter construct regulated by a promoter sequence responsive to AP-1, has been previously described [91]. p18URRL, a luciferase reporter construct regulated by the HPV18 viral URR, has been previously described [65] and was kindly provided by Prof. Felix Hoppe-Seyler (German Cancer Research Center, Heidelberg, Germany). pcDNA3 FLAG JNK1a1 (APF) and JNK2a2 (APF) mutants were obtained from Addgene (#13846 and #13761). c-Jun and ΔJunD were kindly provided by Prof. Simon Arthur (University of Dundee). HPV16 E6 siRNA was purchased from Santa Cruz Biotechnology (SCBT) and had the following sequence: 5′-UGUGUACUGCAAGCAACAG-3′. The HPV18 E6 siRNAs were purchased from Dharmacon (GE Healthcare) and had the following sequences: 5′-CUAACACUGGGUUAUACAA-3′ and 5′-CTAACTAACACTGGGTTAT-3′. The HPV16 and HPV18 E7 siRNA were as previously described and were a kind gift from Prof. Eric Blair (University of Leeds) [92, 93]. siRNA targetting c-Jun were purchased from Qiagen (FlexiTube GeneSolution GS3725 for JUN; SI03077599, SI00034678, SI00034671, SI00034664). The small molecule inhibitors JNK-IN-8 (covalent, irreversible JNK inhibitor), SP600125 (reversable JNK inhibitor), U0126 (MEK1/2 inhibitor) and SB203580 (p38 inhibitor) were purchased from Calbiochem. Human recombinant EGF (236-EG) and HB-EGF (259-HE) were purchased from R&D Systems. Human anti-EGF (MAB236-SP), human anti-HB-EGF (AF-259-SP) and anti-mouse IgG1 control (MAB002) antibodies were purchased from R&D Systems.

### Transfections and mammalian cell lysis

Transfection of plasmid DNA was performed with a DNA to Lipofectamine® 2000 (ThermoFisher) ratio of 1:2.5. 48 hr post transfection, cells were lysed in lysis buffer for western blot analysis, or reseeded into new plates for growth curve analysis, colony formation assays or soft agar assays. Transfection of siRNA was performed with a siRNA to Lipofectamine® 2000 ratio of 1:2. 72-h post transfection cells were lysed in lysis buffer for western blot analysis, or reseeded into new plates for growth curve analysis, colony formation assays or soft agar assays.

### Luciferase reporter assays

Cells seeded into 12 well dishes were transfected with reporter plasmids expressing firefly luciferase under the control of an AP-1 responsive element using PEI or Lipofectamine 2000. Where appropriate, cells were co-transfected with a plasmid of interest, or treated as described in the figure legend. To normalise for transfection efficiency, a pRLTK *Renilla* luciferase reporter plasmid was added to each transfection. After 24 h, samples were lysed in passive lysis buffer (Promega, USA) and activity measured using a dual-luciferase reporter assay system (Promega) as described [91].

### Western blot analysis

Equal amounts of protein from cell lysates were separated by SDS PAGE and transferred onto a nitrocellulose membrane by a semi-dry transfer method (Trans Blot® SD Semi-Dry Transfer cell, Bio-Rad, USA). Membranes were blocked with 5% milk solution before incubation with primary antibodies at 1:1000 dilution unless otherwise stated: Phospho-SAPK/JNK (Thr183/Tyr185) (81E11; 4668, CST), SAPK/JNK (9252, CST), Phospho-ERK1/2 (Thr202/Tyr204) (43705, CST), ERK1/2 (137F5; 4695, CST), Phospho-MAPKAP2 (Thr334) (3007, CST), MAPKAP2 (12155, CST), Phospho-c-Jun (Ser73) (D47G9; 3270, CST), c-Jun (60A8; 9165, CST), JunD (D17G2; 5000, CST), EGFR (E235; ab32077), Abcam) HPV 16/18 E6 (CBT; sc-460), HPV 16 E7 (SCBT; sc-1587), HPV 18 E7 (8E2; ab100953, Abcam), PARP-1 (9542, CST) FLAG (F1804, Sigma-Aldrich), GFP (B-2; sc-9996, Santa Cruz Biotechnology (SCBT)) and GAPDH (SCBT; sc365062) (1:5000) as a loading control. Horseradish peroxidase (HRP)-conjugated secondary antibodies (Sigma-Aldrich, USA) were used at a 1:5000 dilution. Proteins were detected using WesternBright ECL (Advansta, USA) and visualised on X-ray film.

### Chromatin immunoprecipitation

HeLa and CaSKi cells were treated with JNK-IN-8 or transfected with a pool of c-Jun specific siRNA for the required incubation time. Cells were processed for ChIP analysis as previously described [94]. In brief, cells were fixed in 1% formaldehyde for 10 min at room temperature, quenched in 0.25 M glycine, and washed in ice-cold PBS. Cells were harvested by scraping and then lysed in cell lysis buffer (10 mM Tris-HCl, pH 8.0, 10 mM NaCl, 0.2% NP-40, 10 mM sodium butyrate, 50 μg/ml phenylmethylsulfonyl fluoride (PMSF), 1× complete protease inhibitor). Nuclei were collected by centrifugation at 2500 rpm at 4 °C and resuspended in nuclear lysis buffer (50 mM Tris-HCl, pH 8.1, 10 mM EDTA, 1% SDS, 10 mM sodium butyrate, 50 μg/ml PMSF, 1× complete protease inhibitor). Extracted chromatin was then sonicated and chromatin concentration was determined. Approximately 100 µg of chromatin from each sample was used for the experiment. c-Jun was immunoprecipitated using a ChIP grade anti-c-Jun antibody (60A8; 9165, CST). A/G magnetic beads were used to pull down the antibody-chromatin complex. To show antibody specificity, each of the samples was pulled down with an IgG isotype control. The immunoprecipitated chromatin was then processed for quantitative PCR (qPCR); the primer sequences used are available on request. Fold-enrichment compared to negative control IgG isotype control was calculated as in [95].

### RNA extraction, cDNA synthesis and quantitative real Time-PCR

RNA extraction for qRT-PCR was performed using an E.Z.N.A Total RNA Kit I (Omega bio-tek, USA). cDNA was synthesised with 1 µg of input RNA and iScript cDNA synthesis kit (Bio Rad, USA). qRT-PCR was performed on the synthesised cDNA on a Corbett Rotor-Gene 6000 using QuantiFast SYBR Green PCR kit (Qiagen, USA) and analysed using the ΔΔCT method [96] normalised to the *U6* housekeeping gene. Primer sequences are available on request.

### Colony formation assay

Forty-eight or 72-h post-treatment or transfection, cells were trypsinised and reseeded in a six-well plate at 500 cells per well and left to incubate for 14–21 days. Colonies were then stained (1% crystal violet, 25% methanol) and colonies were counted manually. Each experiment was repeated a minimum of three times.

### Soft agar assay

Cells were treated or transfected as required. Sixty millimetre dishes were coated with a layer of 1% agarose (ThermoFisher Scientific) in 2× DMEM (ThermoFisher Scientific) supplemented with 20% FBS. Forty-eight hours post-transfection, cells were trypsinised and added to 0.7% agarose in 2× DMEM (ThermoFisher Scientific) supplemented with 20% FBS at 1000 cells/mL. Once set, DMEM supplemented with 10% FBS and 50 U/mL penicillin was added. The plates were then incubated for 14–21 days. Each experiment was repeated at least three times. Visible colonies were counted manually.

### Flow cytometry

Cells were treated or transfected as required. 48 or 72-h post-transfection, cells were harvested and fixed in 70% ethanol overnight. The ethanol was removed, and cells washed with PBS containing 0.5% (w/v) BSA. Cells were stained with PBS containing 0.5% BSA, 50 μg/mL propidium iodide (Sigma-Aldrich) and 5 μg/mL RNase (Sigma-Aldrich) and incubated for 30 min at room temperature. Samples were processed on a CytoFLEX S flow cytometer (Beckman Coulter) and analysed using CytExpert (Beckman Coulter).

### Annexin V assay

Annexin V apoptosis assay (TACS Annexin V kit; 4830-250-K) was performed as indicated on the product datasheet. In brief, cells seeded in 6-well plates were treated or transfected as required. Cells were trypsinised and collected by centrifugation. 1 × 10^6^ cells were then incubated in 100 μL Annexin V reagent (10 μL 10× binding buffer, 10 μL propidium iodide, 1 μL Annexin V-FITC (diluted 1 in 500) and 79 μL ddH2O) for 15 min at room temperature in the dark. Samples were diluted in 1× binding buffer before analysis by flow cytometry. Samples were processed on a CytoFLEX S flow cytometer (Beckman Coulter) and analysed using CytExpert (Beckman Coulter).

### TCPA data analysis

RPPA data from the Cancer Genome Atlas (TCGA) was downloaded via The Cancer Proteome Atlas (TCPA) online repository [37, 38]. Correlation between Phospho-SAPK/JNK (Thr183/Tyr185), Phospho-ERK1/2 (Thr202/Tyr204) and Phospho-p38 (Thr180/Tyr182) with the substrate Phospho-c-Jun (Ser73) was assessed.

### Statistical analysis

Data points from at least three individual, biological repeats are shown in each graph. Unless otherwise indicated in the figure legends, data were analysed using a two-tailed, unpaired Student’s *t*-test and graphs were prepared using the GraphPad Prism software (GraphPad, USA). Error bars represent the mean +/− standard deviation. Statistical significance was determined as follows: *P < 0.05, **P < 0.01, ***P < 0.001. Survival analysis was performed using the Kaplan–Meier method with the log-rank test (univariate).

### Funding

This work was supported by Medical Research Council (MRC) funding to AM (MR/ K012665 and MR/S001697/1). ELM has received support from a Wellcome Trust studentship (1052221/Z/14/Z) and the Wellcome Institutional Strategic Support Fund (ISSF) (204825/Z/16/Z). MRP is funded by Biotechnology and Biological Sciences Research Council studentships (BB/M011151/1). JAS is funded by a Faculty of Biological Sciences, University of Leeds scholarship. The funders had no role in study design, data collection and analysis, decision to publish, or preparation of the manuscript.

## Supplementary information

Supplementary Figure Legends

Supplementary Figure 1

Supplementary Figure 2

Supplementary Figure 3

Supplementary Figure 4

Supplementary Figure 5

Supplementary Figure 6

Supplementary Figure 7

Supplementary Figure 8
