# Supplementary material for: E6-mediated activation of JNK drives EGFR signalling to promote proliferation and viral oncoprotein expression in cervical cancer
Source: Cell Death Differ. 2020 Dec 10;28(5):1669–87. doi: 10.1038/s41418-020-00693-9 (PMC8166842; doi:10.1038/s41418-020-00693-9)
Supplement: Supplementary file 1 — Supplementary Figure Legends [file 41418_2020_693_MOESM1_ESM.docx]

**Supplementary Figure 1.** **The JNK substrates c-Jun and JunD are up-regulated in HPV+ cervical cancer. A)** qPCR analysis of *JUNB*, *JUN*, and *JUND* expression in normal human keratinocytes (NHK) and NHKs containing the HPV18 genomes (HPV18). *U6* served as a loading control. **B)** Representative western blot of normal human keratinocytes (NHK) and NHKs containing the HPV18 genomes (HPV18) analysed for phosphorylated and total c-Jun and JunD. GAPDH served as a loading control. Data shown is representative of at least three independent experiments. **C)** qPCR analysis of *JUNB*, *JUN*, and *JUND* expression in six cervical cancer cell lines – two HPV- (C33A and Dotc2 4510), two HPV16+ (SiHa and CaSKi) and HPV18+ (SW756 and HeLa) – and NHK cells. *U6* served as a loading control. **D)** Representative western blot analysis of a panel of HPV- and HPV+ cervical cancer cell lines and NHK cells for phosphorylated and total c-Jun and JunD. GAPDH served as a loading control. Data shown is representative of at least three independent experiments. **E)** Kaplan Meier analysis of overall survival in cervical cancer based on *JUN* and *JUND* expression from TCGA data. Error bars represent the mean +/- standard deviation of a minimum of three biological repeats. *P<0.05, **P<0.01, ***P<0.001 (Student’s t-test).

**Supplementary Figure 2. JNK inhibitor SP600125 inhibits JNK activity in HPV+ cervical cancer cells. A)** Representative western blots of HeLa and CaSKi cells treated with increasing doses of SP600125. Lysates were analysed for the phosphorylation and expression of the JNK substrate c-Jun and JunD. GAPDH was used as a loading control. Data shown is representative of at least three independent experiments.

**Supplementary Figure 3. The JNK isoforms JNK1 and JNK2 are required for proliferation in HPV+ cervical cancer. A)** Representative western blots of HeLa and CaSKi cells transiently transfected with dominant negative forms of JNK1 and JNK2. Lysates were analysed for the phosphorylation and expression of the JNK substrate c-Jun. Expression of dominant negative forms of JNK1 and JNK2 was confirmed using anti-FLAG antibodies. GAPDH was used as a loading control. Data shown is representative of at least three independent experiments. **B)** Growth curve analysis of HeLa and CaSKi cells transiently transfected with dominant negative forms of JNK1 and JNK2. **C)** Colony formation assay (anchorage dependent growth) of HeLa and CaSKi cells transiently transfected with dominant negative forms of JNK1 and JNK2. **D)** Soft agar assay (anchorage independent growth) of HeLa and CaSKi cells transiently transfected with dominant negative forms of JNK1 and JNK2. Error bars represent the mean +/- standard deviation of a minimum of three biological repeats. *P<0.05, **P<0.01, ***P<0.001 (Student’s t-test).

**Supplementary Figure 4. JNK inhibition has minimal impact on the proliferation of HPV- cervical cancer cells. A)** Representative western blots of C33A cells treated with increasing doses of JNK-IN-8. Lysates were analysed for the phosphorylation and expression of the JNK substrate c-Jun. GAPDH was used as a loading control. Data shown is representative of at least three independent experiments. **B)** Growth curve analysis of C33A cells treated with JNK-IN-8 (3 µM). **C)** Colony formation assay (anchorage dependent growth) of C33A cells treated with JNK-IN-8 (3 µM). **D)** Soft agar assay (anchorage independent growth) of C33A cells treated with JNK-IN-8 (3 µM). Error bars represent the mean +/- standard deviation of a minimum of three biological repeats. *P<0.05, **P<0.01, ***P<0.001 (Student’s t-test).

**Supplementary Figure 5. c-Jun over-expression enhances proliferation in HPV+ cervical cancer cells. A)** Representative western blots of HeLa and CaSKi cells over-expressing c-Jun. Lysates were analysed for the c-Jun expression. GAPDH was used as a loading control. Data shown is representative of at least three independent experiments. **B)** Growth curve analysis of HeLa and CaSKi cells over-expressing c-Jun. **C)** Colony formation assay (anchorage dependent growth) of HeLa and CaSKi cells over-expressing c-Jun. **D)** Soft agar assay (anchorage independent growth) of HeLa and CaSKi cells over-expressing c-Jun. Error bars represent the mean +/- standard deviation of a minimum of three biological repeats. *P<0.05, **P<0.01, ***P<0.001 (Student’s t-test).

**Supplementary Figure 6. Inhibition of AP-1 activity inhibits proliferation in HPV+ cervical cancer cells. A)** Representative western blots of HeLa and CaSKi cells transfected with a JunD mutant that lacks the transactivation domain (ΔJunD). Lysates were analysed for the expression of ΔJunD. GAPDH was used as a loading control. Data shown is representative of at least three independent experiments. **B)** Luciferase reporter assay from HeLa cells co-transfected with an AP-1 luciferase reporter and ΔJunD. Promoter activity was measured using a dual-luciferase system. Data are presented as relative to the GFP transfected control. **C)** Growth curve analysis of HeLa and CaSKi cells transfected with ΔJunD. **D)** Colony formation assay (anchorage dependent growth) of HeLa and CaSKi cells transfected with ΔJunD. **E)** Soft agar assay (anchorage independent growth) of HeLa and CaSKi cells transfected with ΔJunD. Error bars represent the mean +/- standard deviation of a minimum of three biological repeats. *P<0.05, **P<0.01, ***P<0.001 (Student’s t-test).

**Supplementary Figure 7.** **Inhibition of JNK activity, or knock down of c-Jun, induces G2/M phase accumulation and apoptosis. A)** Flow cytometric analysis of cell cycle profile of HeLa and CaSKi cells treated with JNK-IN-8 (3 µM) or SP600125 (10 µM)**. B)** Flow cytometric analysis of Annexin V assay in HeLa and CaSKi cells treated with JNK-IN-8 (3 µM) or SP600125 (10 µM). **C)** Representative western blot of HeLa and CaSKi cells treated with JNK-IN-8 (3 µM) or SP600125 (10 µM) analysed for PARP cleavage. GAPDH served as the loading control. Data shown is representative of at least three independent experiments. **D)** Flow cytometric analysis of cell cycle profile of HeLa and CaSKi cells after transfection of a pool of siRNA against the JNK substrate c-Jun. **E)** Flow cytometric analysis of Annexin V assay in HeLa and CaSKi cells after transfection of a pool of siRNA against the JNK substrate c-Jun. **F)** Representative western blot of HeLa and CaSKi cells after transfection of a pool of siRNA against the JNK substrate c-Jun and analysed for PARP cleavage. GAPDH served as the loading control. Data shown is representative of at least three independent experiments. Error bars represent the mean +/- standard deviation of a minimum of three biological repeats. *P<0.05, **P<0.01, ***P<0.001.

**Supplementary Figure 8. EGF activates JNK/Jun signalling in HPV+ cervical cancer cells. A)** Representative western blots of HeLa cells treated with EGF (50 ng/mL) for different amounts of time. Lysates were analysed for phosphorylated and total EGFR, phosphorylated and total JNK, phosphorylated and total c-Jun and total JunD. GAPDH served as a loading control. Data shown is representative of at least three independent experiments.
