# Supplementary figures and images for: E6-mediated activation of JNK drives EGFR signalling to promote proliferation and viral oncoprotein expression in cervical cancer
Source: Cell Death Differ. 2020 Dec 10;28(5):1669–87. doi: 10.1038/s41418-020-00693-9 (PMC8166842; doi:10.1038/s41418-020-00693-9)

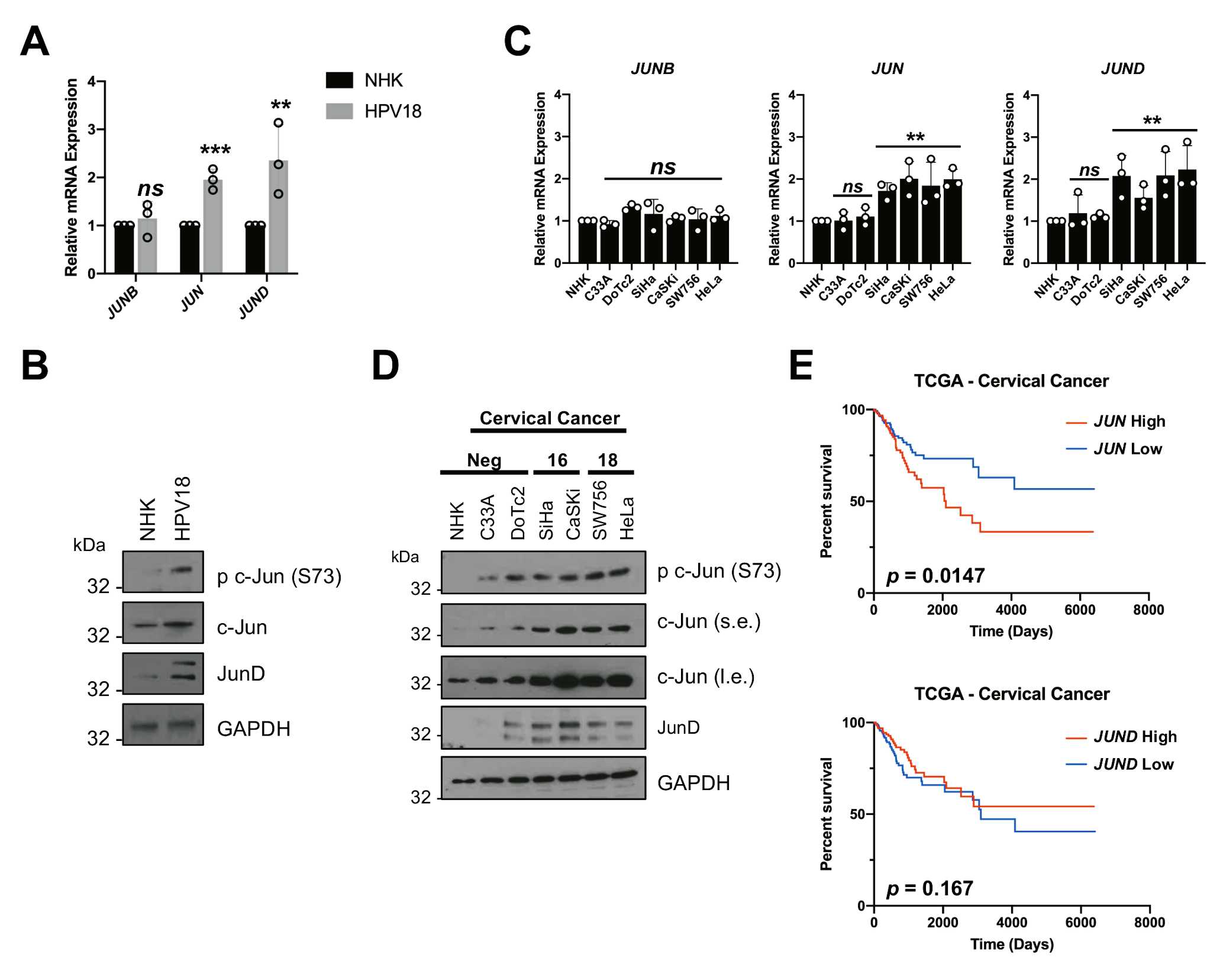

Supplement: Supplementary file 2 — Supplementary Figure 1 [file 41418_2020_693_MOESM2_ESM.tif]

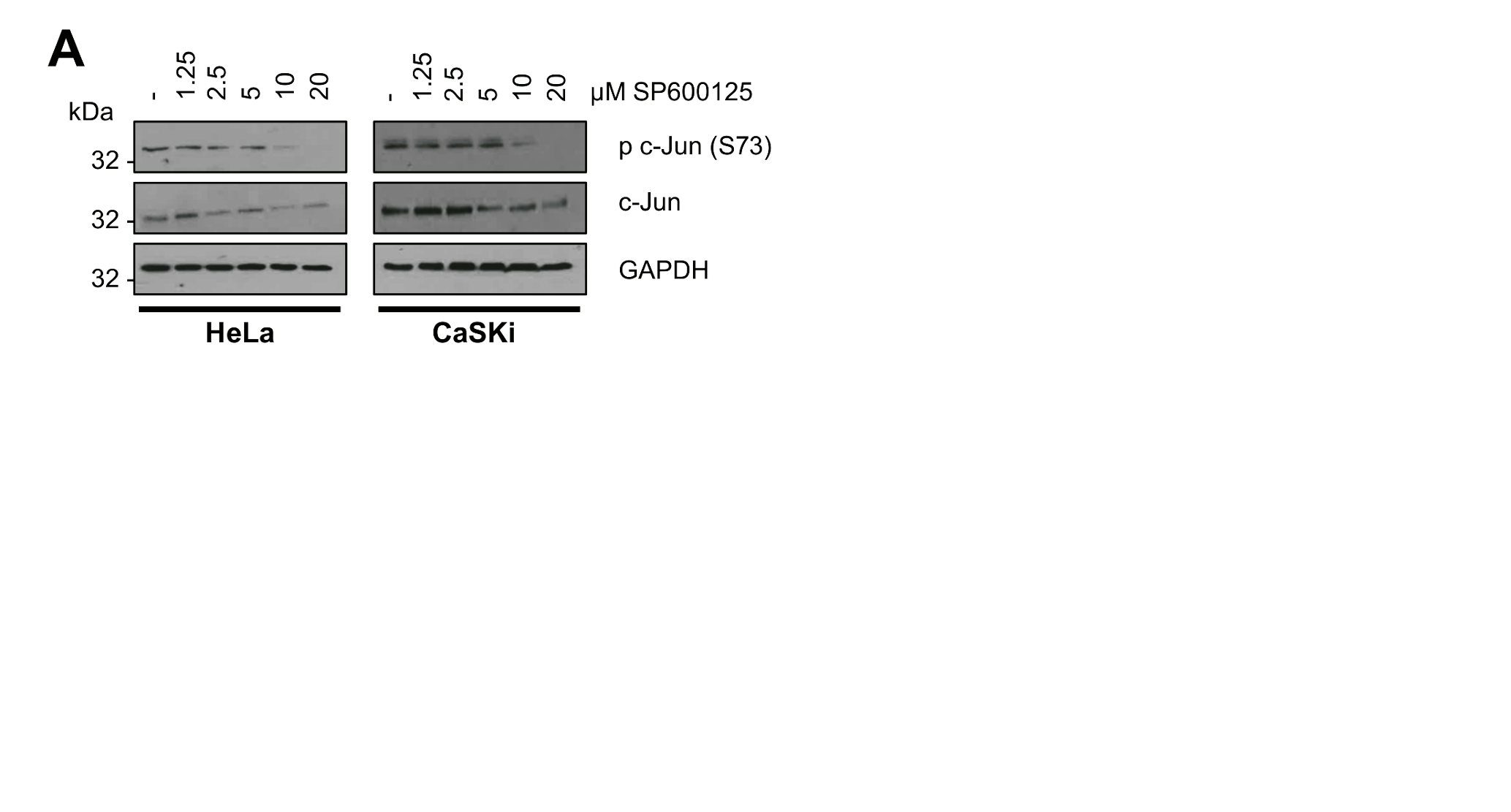

Supplement: Supplementary file 3 — Supplementary Figure 2 [file 41418_2020_693_MOESM3_ESM.tif]

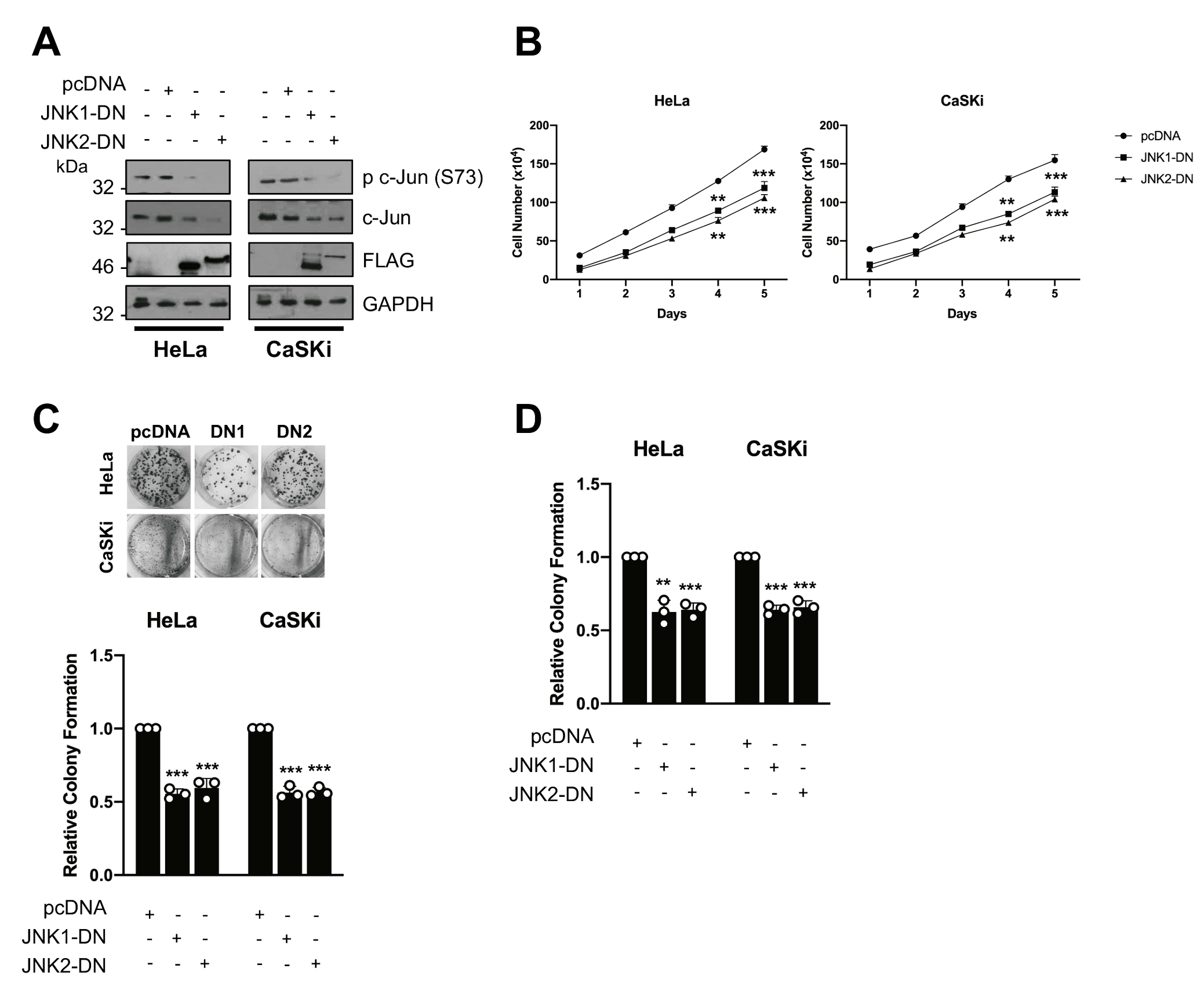

Supplement: Supplementary file 4 — Supplementary Figure 3 [file 41418_2020_693_MOESM4_ESM.tif]

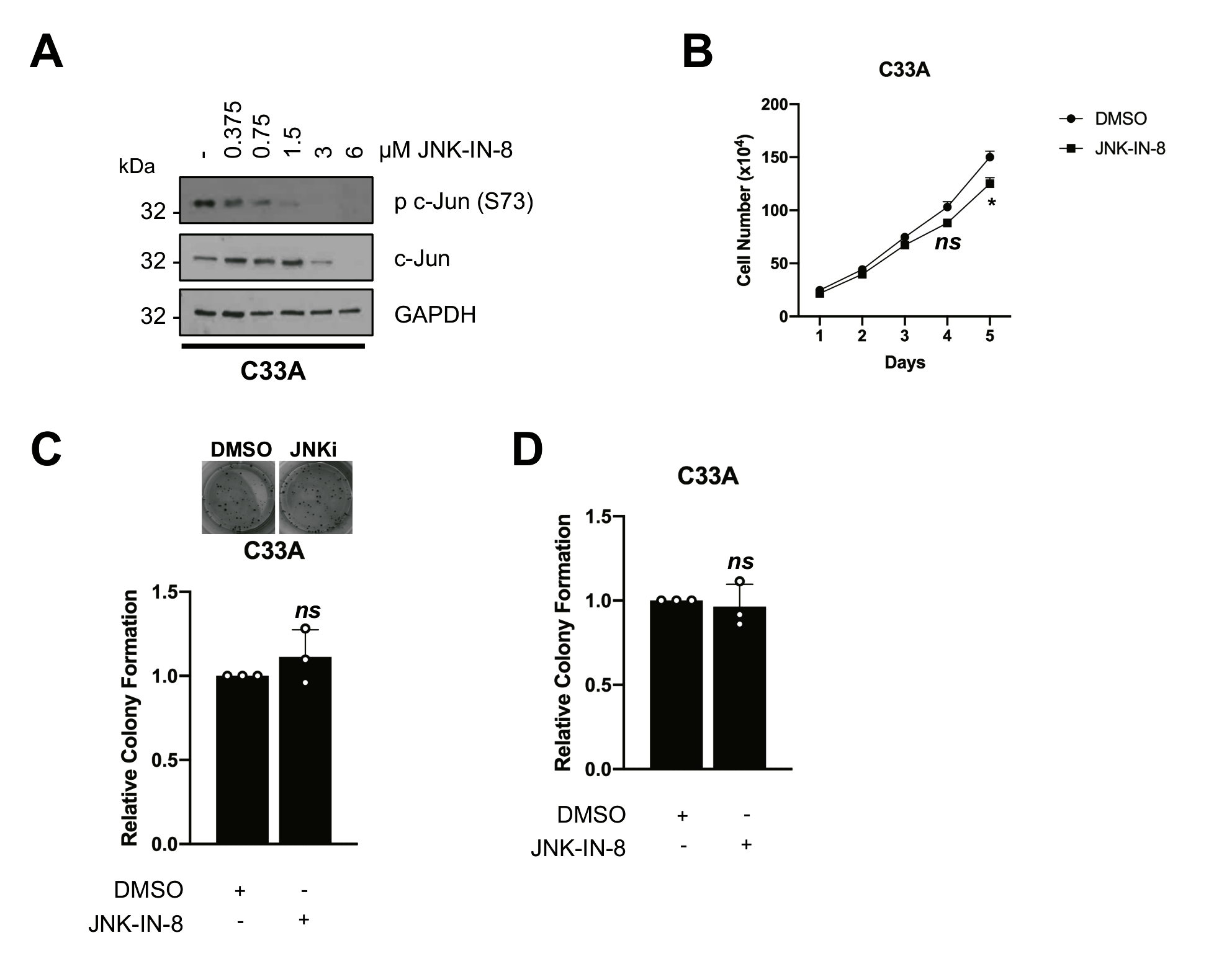

Supplement: Supplementary file 5 — Supplementary Figure 4 [file 41418_2020_693_MOESM5_ESM.tif]

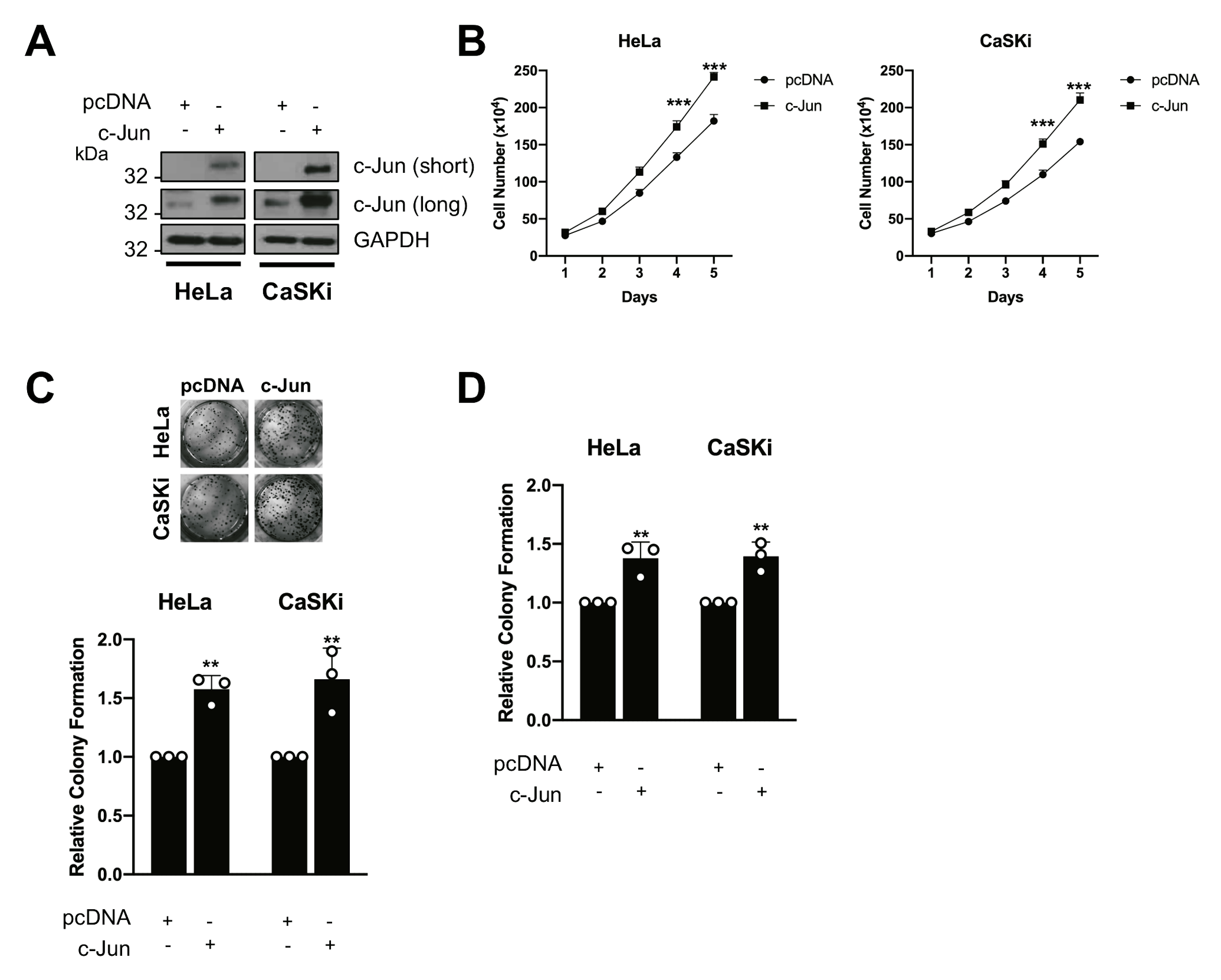

Supplement: Supplementary file 6 — Supplementary Figure 5 [file 41418_2020_693_MOESM6_ESM.tif]

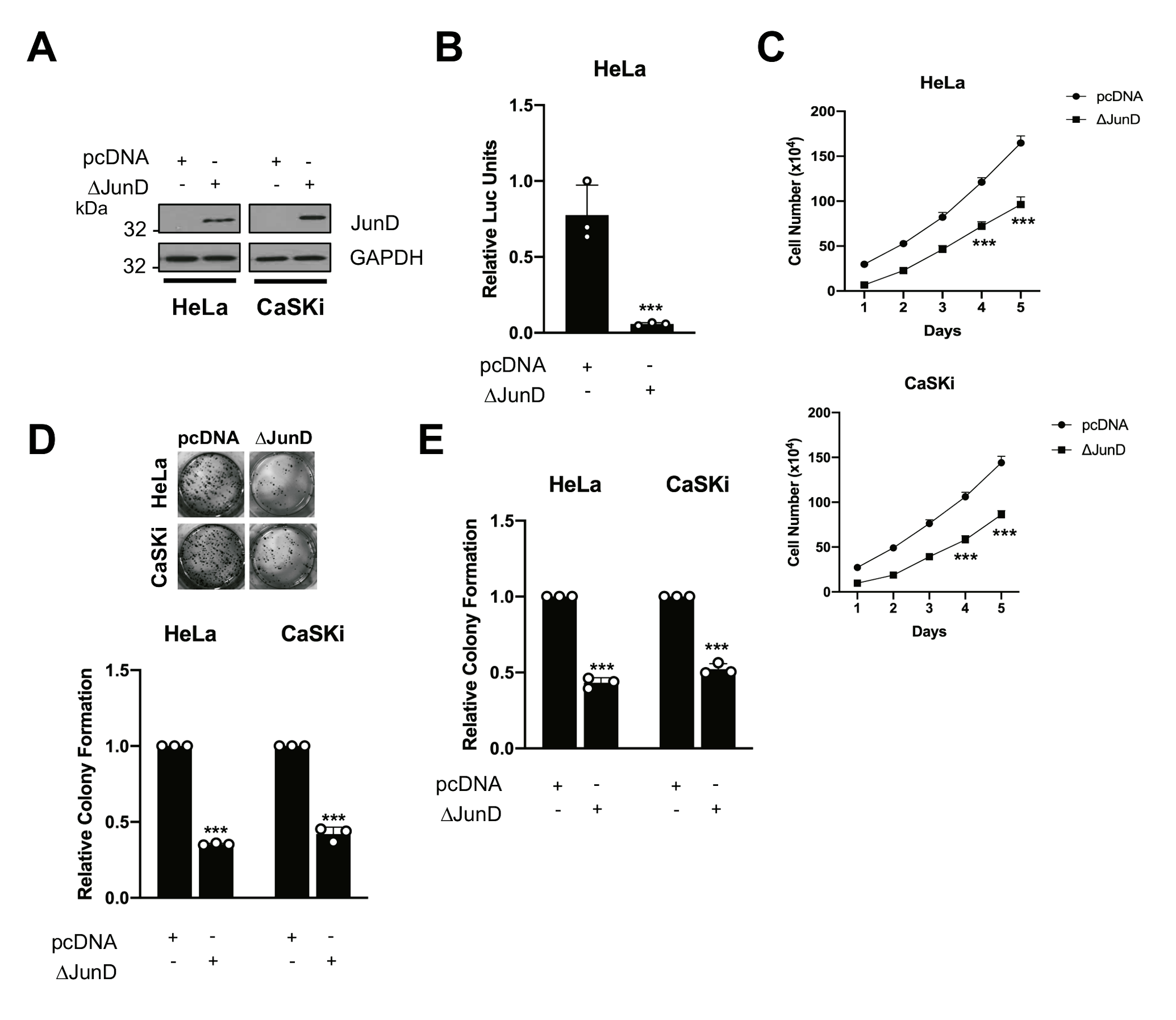

Supplement: Supplementary file 7 — Supplementary Figure 6 [file 41418_2020_693_MOESM7_ESM.tif]

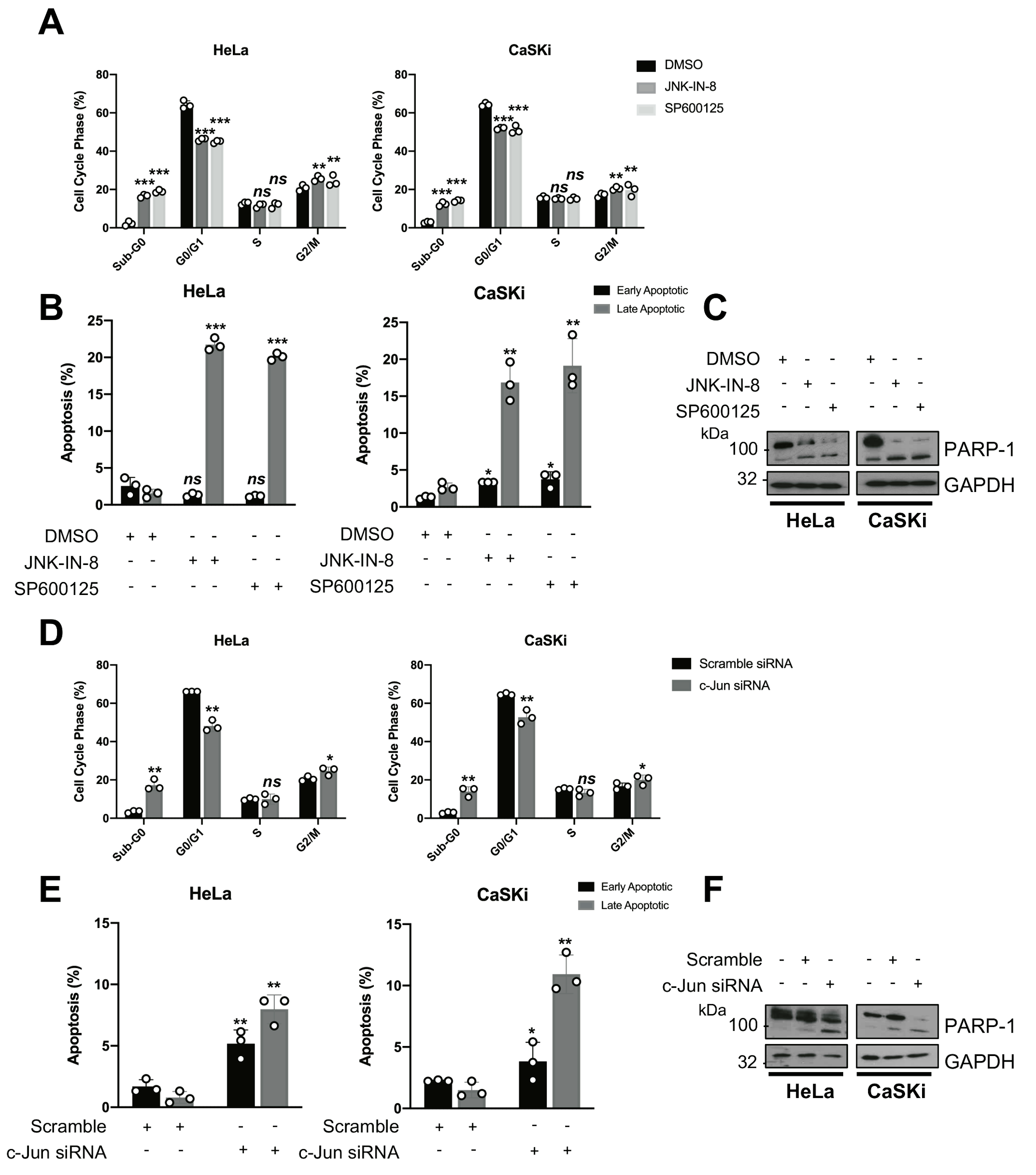

Supplement: Supplementary file 8 — Supplementary Figure 7 [file 41418_2020_693_MOESM8_ESM.tif]

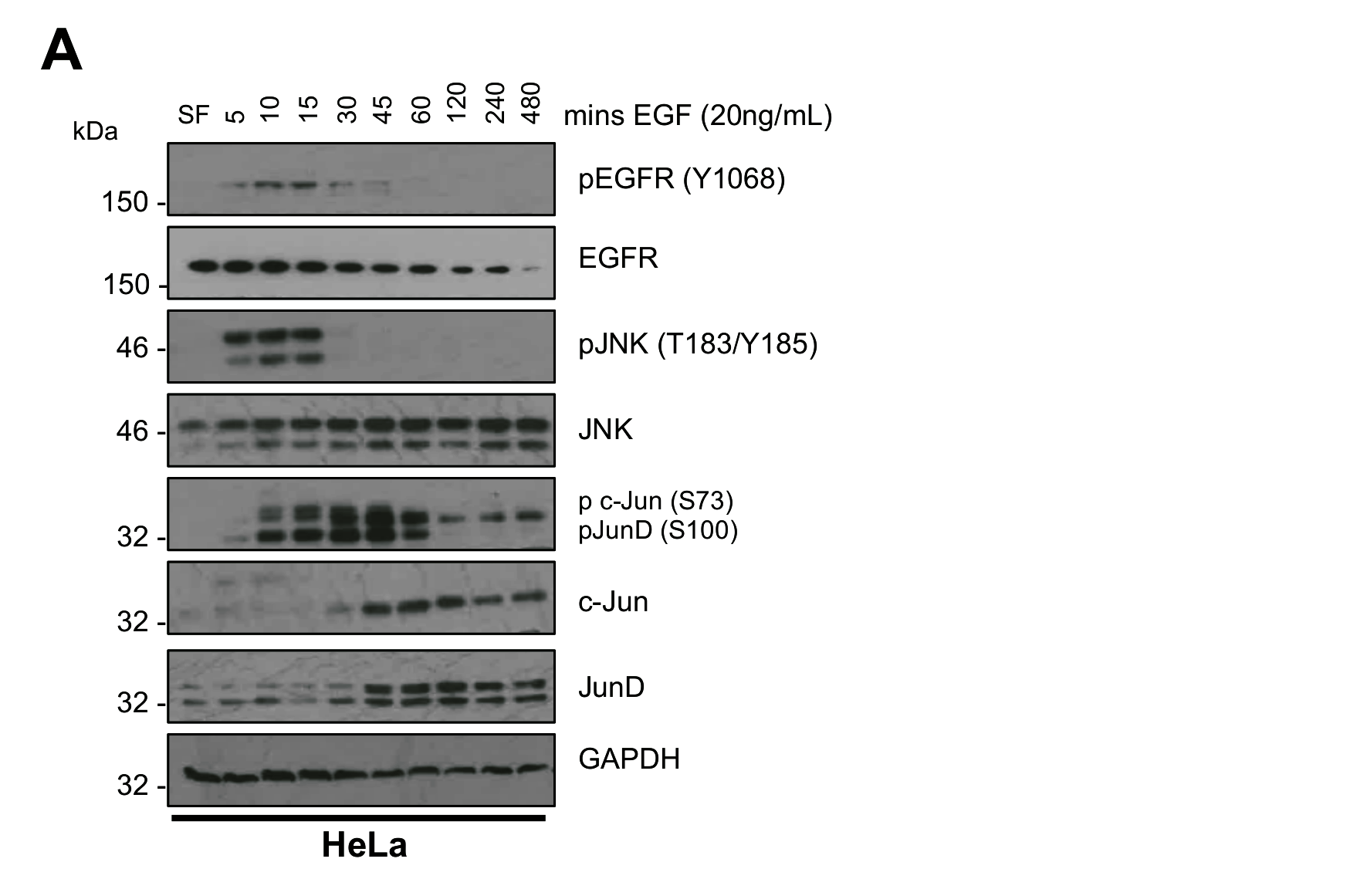

Supplement: Supplementary file 9 — Supplementary Figure 8 [file 41418_2020_693_MOESM9_ESM.tif]
